# Mediators of the Pain–Depression Relationship in Adults with Chronic Pain: A Systematic Review and Exploratory Meta-Analyses

**DOI:** 10.3390/jcm15155784

**Published:** 2026-07-23

**Authors:** Michael Tenti, Stefania Proietti, Corrado Fagnani, Emanuela Medda, Letizia Sampaolo, Laura Camoni, Federica Cilenti, Giorgia Varallo, Valentina Malafoglia, William Raffaeli, Virgilia Toccaceli

**Affiliations:** 1 ISAL Foundation-ETS, Institute for Research on Pain, 47921 Rimini, Italy; 2 Agea Coordinating Body, 00185 Rome, Italy; 3 Centre for Behavioural Sciences and Mental Health, Istituto Superiore di Sanità (Italian National Institute of Health), 00161 Rome, Italy; corrado.fagnani@iss.it (C.F.); emanuela.medda@iss.it (E.M.); laura.camoni@iss.it (L.C.); virgilia.toccaceli@iss.it (V.T.); 4 Centre for Disease Prevention and Health Promotion, Istituto Superiore di Sanità (Italian National Institute of Health), 00161 Rome, Italy; 5 Department of Biomedical, Metabolic and Neural Sciences, University of Modena and Reggio Emilia, 41125 Modena, Italy; g.varallo@unimore.it; 6 IRCCS San Raffaele Roma, 00166 Rome, Italy

**Keywords:** systematic review, meta-analysis, mediation analysis, chronic pain, pain intensity, depression, depressive symptoms, helplessness, pain catastrophizing

## Abstract

**Background/Objectives**: Pain intensity and depressive symptoms are closely associated in adults with chronic pain (CP), contributing to greater disability, poorer treatment outcomes, and increased healthcare costs. However, the mechanisms underlying this symptom-level relationship remain incompletely understood. This systematic review and meta-analysis aimed to (i) identify all candidate factors mediating the effect of pain intensity on depressive symptoms, or vice versa, in adults with CP and (ii) estimate the magnitude of indirect, direct and total effects reported across eligible studies. **Methods**: Seven databases were searched in May–July 2023 and updated in May–June 2024. Observational studies and randomized controlled trials evaluating mediators of the relationship between pain intensity and depressive symptoms, or vice versa, in adults with CP were eligible. Data extraction and methodological appraisal were performed independently by three reviewers. Findings were synthesized narratively using vote counting and displayed using harvest plots. When appropriate, meta-analyses were conducted using regression coefficients, standard errors and sample sizes to estimate pooled indirect, direct and total effects. **Results**: The search identified 6826 studies, of which 34 (47 mediation models) met the inclusion criteria. Twenty-nine studies (combined *n* = 13,587) examined the effect of pain intensity on depressive symptoms, identifying 24 candidate mediators. Exploratory meta-analyses were feasible only for pain catastrophizing (indirect effect [IE]: β = 0.150; 95% confidence interval [CI]: 0.066, 0.233) and helplessness (IE: β = 0.101; CI: 0.066, 0.137). The pooled estimate for pain catastrophizing showed substantial heterogeneity and was highly influenced by a single study. Sleep quality, pain self-efficacy and pain interference showed preliminary evidence of mediation in narrative synthesis. Nine studies (combined *n* = 1359) investigated the effect of depressive symptoms on pain intensity, identifying 12 candidate mediators. Pain catastrophizing was the most consistently supported mediator, although quantitative synthesis was not feasible. **Conclusions**: Several potentially modifiable mediators of the pain–depression relationship were identified and may represent targets for multidisciplinary interventions. However, quantitative findings remain preliminary because of the limited number of studies, substantial heterogeneity for some mediators, and the predominance of cross-sectional evidence. Robust three-wave longitudinal mediation studies with adequate statistical power, standardized measures and reporting, and theory-driven models are needed to establish the temporal validity of candidate mediators and strengthen causal inferences.

## 1. Introduction

Chronic pain (CP), defined as pain persisting or recurring for longer than 3 months [1], encompasses a heterogenous group of conditions, including chronic low back pain, fibromyalgia, and migraine [2,3]. It affects about 20% of the worldwide population [4,5,6], is one of the leading causes of years lived with disability [7,8], and imposes a heavy burden on all aspects of patients’ lives [9]. CP is also associated with mental comorbidities that worsen the clinical picture [10,11,12]. In particular, depressive symptoms are highly prevalent in individuals with CP, occurring more commonly in patients with CP than in the general population [6,10,13,14,15]. When these two conditions co-occur, pain intensity and depressive symptoms negatively influence each other, leading to an increase in the severity and number of symptoms, greater functional impairment, reduced quality of life, poorer response to treatments and higher healthcare and social costs [16,17,18].

**Figure 1:**
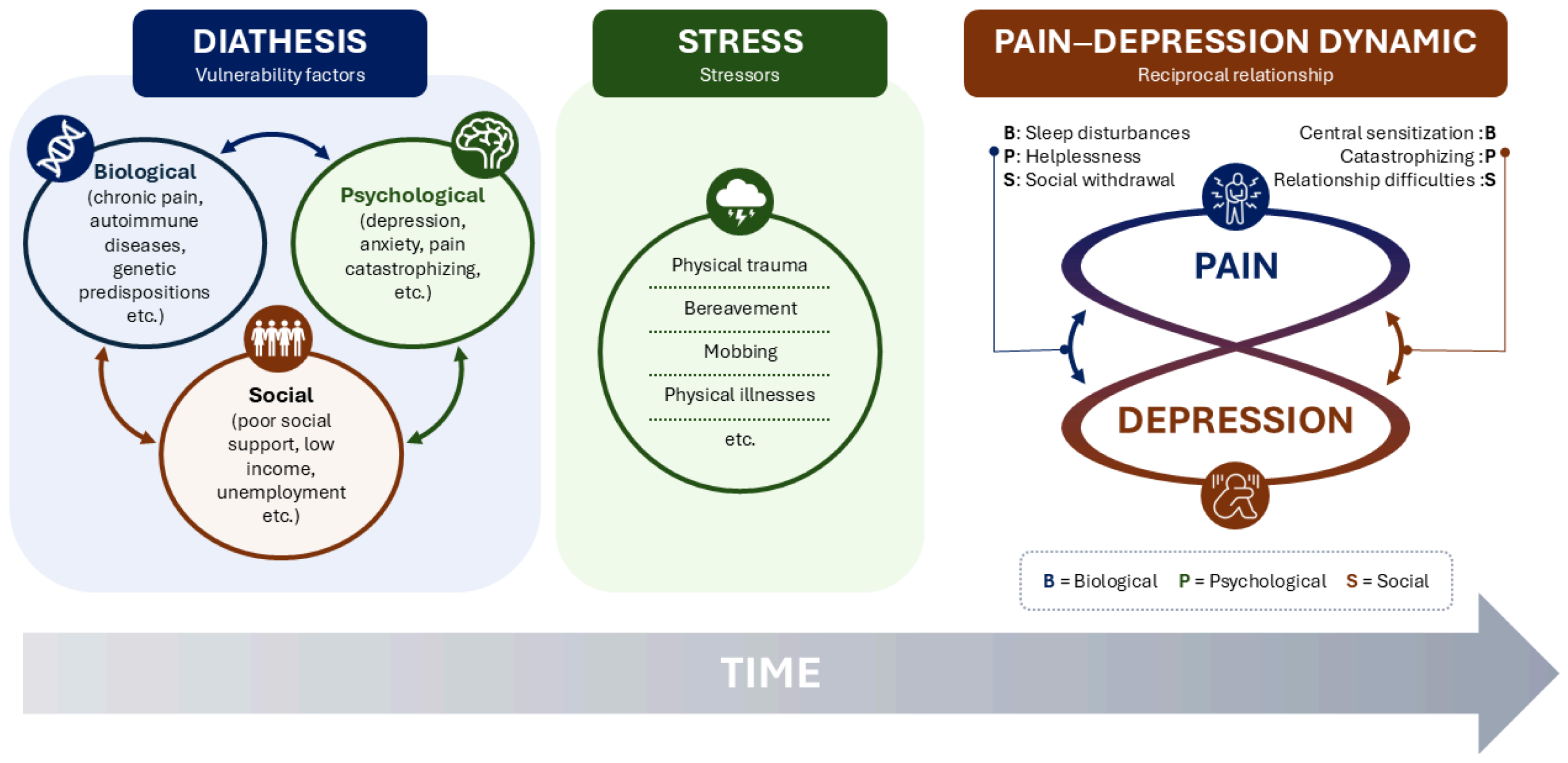
Conceptualization of pain and depression development and reciprocal influence within the Biopsychosocial Diathesis–Stress Model.

Recent evidence points to a bidirectional relationship between pain and depression [19,20,21,22,23,24]. For example, in a large prospective cohort study (*n* = 504,365), a pre-existing, persistent pain disorder at baseline predicted the onset of depression to the same degree that baseline depression predicted the subsequent onset of persistent pain [25]. Similarly, at symptom level, a longitudinal study in patients with psoriatic arthritis found reciprocal associations between pain intensity and depressive symptoms over time [22]. These findings are in line with the biopsychosocial Diathesis–Stress Model, already proposed and tested in the field of CP [26,27,28,29,30,31]. According to this model, different predisposing factors (either biological or psychosocial, including pain or depressive symptoms) may interact with each other, representing individual vulnerability (diathesis) to an illness. Certain environmental factors or life events (stressors such as trauma and bereavement) may activate an individual’s vulnerability, precipitating illnesses, be it pain or depression. Once a clinical condition is established, pain intensity and depressive symptoms may then mutually influence each other, contributing to the continuation and potential worsening of both symptom domains over time (Figure 1).

In this context, understanding the mechanisms by which pain intensity and depressive symptoms influence each other in individuals with CP is more informative than determining the directionality of their relationship. Mediation analysis is particularly useful in this regard, as it examines the extent to which an intermediate variable (i.e., the mediator) explains the effect of an exposure on an outcome [32]. This approach can shed light on the mechanisms by which a factor, either pain intensity or depression symptoms in our case, may affect each other, informing future interventions and treatment targets [33,34,35].

Evidence from mediation studies already showed that different constructs may exert a significant influence on the pain intensity–depressive symptoms relationship in individuals with CP. For example, reductions in perceived control and personal mastery have been implicated in the development of depressive symptoms in patients with CP [36], whereas restricted social participation and low satisfaction with social participation were found to mediate the effect of pain intensity on depressive symptoms in individuals with CP with spinal cord injury [37]. From a bidirectional perspective, pain catastrophizing and illness perception were found to mediate the link between depressive symptoms and pain intensity, respectively, in patients with irritable bowel syndrome [38] and rheumatoid arthritis [39].

Although the bidirectional association between pain and depression at symptom level (hereafter referred to as the “pain–depression” relationship) has been extensively documented in the context of CP, the mechanisms mediating this relationship remain poorly understood and have never been comprehensively synthesized. To bridge this gap, we conducted the first systematic review of mediation studies investigating the mechanisms underlying the bidirectional relationship between pain intensity and depressive symptoms in adults with CP, with quantitative synthesis whenever feasible. Specifically, we aimed to (i) identify all candidate factors mediating the effect of pain intensity on depressive symptoms, or vice versa, in adults with CP and (ii) estimate the magnitude of indirect, direct and total effects reported across eligible studies.

## 2. Materials and Methods

### 2.1. Registration of Systematic Review

This review is reported adhering to (i) the 2020 Preferred Reporting Items for Systematic Reviews and Meta-Analyses (PRISMA) guideline [40] and the Synthesis Without Meta-Analysis (SWiM) extension [41]; (ii) APA’s Meta-Analysis Reporting Methods (MARS) [42]. The completed PRISMA 2020 Checklist and PRISMA 2020 for Abstracts Checklist are provided in Supplementary Files S1 and S2, respectively. The review protocol was registered in the Prospective Register of Systematic Reviews (PROSPERO; ID CRD42023435455, https://www.crd.york.ac.uk/PROSPERO/view/CRD42023435455, last accessed on 20 July 2026.

### 2.2. Search Strategy

Between 9 May 2023 and 6 July 2023, an electronic search was conducted in the following databases/registers: PubMed/Medline, Scopus, Web of Science, CINAHL, Psychology Database, PsycINFO, and Cochrane Central Register of Controlled Trials. The search was updated between 17 May and 10 June 2024 to control for newly published studies during the screening procedure. The search strategy included sets of terms combined with an “AND” Boolean operator, particularly two sets for “pain” and “depression”, and a set already used by other systematic reviews to detect mediation studies [33,35]. The complete search strings employed in each database are presented in Supplementary File S3. Additional relevant studies not identified by the electronic search were identified from the reference lists of the included articles.

### 2.3. Eligibility Criteria

The following inclusion criteria were considered:

- Cross-sectional or longitudinal observational studies or randomized controlled trials.
- Studies involving individuals affected by CP, defined as pain persisting or recurring for at least 3 months [1].
- Studies including a measure of pain intensity as the predictor and a measure of depressive symptoms as the outcome in the mediation analysis, or vice versa.
- Studies conducted on adult (≥18 years) populations.
- Studies including a measure of a putative mediating variable with a formal test of mediation (e.g., causal steps approach, product of coefficient approach) and a test of the significance of mediated effects (e.g., Sobel test or bootstrapped confidence intervals).
- Studies published in English, Italian, or German.
- Studies published from 1 January 2000 onwards in peer-reviewed journals, accessible in full text.

Studies published in English, Italian, or German were considered eligible. This decision was based on exploratory searches, which indicated that the relevant literature was almost exclusively published in English, with only a small number of potentially eligible studies identified in German, together with the availability of reviewers able to assess studies published in these languages. Non-original research, conference proceedings, dissertations, and book chapters were excluded. Studies on non-human populations, children, adolescents, and patients with cancer were also excluded. We included studies using any validated measure of pain intensity or depressive symptoms but excluding those in which pain intensity or depression were combined with other constructs (e.g., “pain severity” as a composite of intensity and interference or the Hospital Anxiety and Depression Scale total score combining anxiety and depression). Consistent with previous reviews [33,35], no restrictions were placed on the type of mediator investigated. Studies evaluating any psychological, behavioral, social, biological, or functional mediator of the relationship between pain intensity and depressive symptoms (or vice versa) were considered eligible. Moreover, we excluded studies on mixed populations in which patients with CP represented less than 75% of the sample [33] or in which it was not possible to ascertain the exact percentage of individuals affected by CP in the sample. Even when pain duration was not an explicit inclusion criterion and was not directly reported, we included studies involving populations with syndromes typically characterized by CP (e.g., osteoarthritis, interstitial cystitis) as well as studies conducted in specific clinical settings (e.g., orthopedic clinics), if available data (e.g., mean and standard deviation) indicated that most participants had experienced pain for ≥3 months.

### 2.4. Selection Process and Data Extraction

An expert in database searching (L.S.) performed the searches and exported the results to the Zotero bibliographic management software. After removing the duplicate references, three independent reviewers (F.C., E.M., and M.T.) used the eligibility criteria to screen the titles/abstracts of the retrieved studies. Articles that could not be deemed eligible from the title/abstract were evaluated in full text by the same reviewers. Any uncertainty or disagreements between reviewers regarding eligibility were discussed with a fourth reviewer (V.T.) until consensus was reached.

For each of the included studies, three reviewers independently extracted the data (E.M., V.M., and S.P.); in the case of any disagreements, two further reviewers (F.C. and V.T.) were consulted to reach a consensus.

As in previous systematic reviews of mediation studies [33,35], the data extraction form used in this study included the following information: study population and its setting; study design; duration of follow-up (if applicable); number of participants at baseline and at follow-up (if applicable); participant characteristics, including mean age and the proportion of females; the instruments used to measure pain intensity and depressive symptomatology; the exposure, mediator and outcome variables examined in formal mediation tests; the mediation tests applied; and the results of these tests.

For studies included in the meta-analysis, two reviewers (G.V. and M.T.) independently extracted unstandardized (B) and standardized (β) regression coefficients, along with their standard errors, for the a and b paths of the mediation model, as well as for the total, direct, and indirect effects. Uncertainties were resolved through discussion with a third reviewer (C.F.) until consensus was reached. In some studies, values were derived indirectly. When the indirect effect was not explicitly reported in the article, it was calculated by multiplying the coefficients of paths a and b (i.e., a × b), both for unstandardized and standardized estimates.

To address comparability issues between standardized and unstandardized coefficients, we followed a previously described methodological approach [43]. Accordingly, the corresponding procedures were applied to account for differences in scaling and to ensure consistent handling of both reported and derived indirect effects. When the standard error (SE) was not available but the 95% confidence interval (CI95%) was reported, the inverse formula was applied to calculate the desired measure. When essential statistical information was not available in the publication, up to three attempts were made to contact the corresponding author by e-mail to request the missing data.

### 2.5. Methodological Appraisal of Mediation Studies

Consistent with previous reviews of mediation studies [33,35,44], the methodological appraisal of the included mediation models was conducted using an adapted version of the critical appraisal tool developed by Mansell and colleagues [45]. This instrument is specifically designed to appraise key methodological aspects of mediation analyses and should not be interpreted as a comprehensive risk-of-bias assessment. The adapted instrument consists of eight dichotomous (yes/no) criteria evaluating methodological features of mediation studies. Each item was scored as present (1) or absent (0), resulting in an appraisal total score ranging from 0 to 8. The eight criteria were (Q1) a theoretical or statistical rationale for the mediation hypothesis; (Q2) clarity of study aims; (Q3) adequacy of sample size; (Q4) psychometric quality of measures; (Q5) appropriateness of the statistical methods for mediation analysis; (Q6) temporal precedence of exposure over mediator; (Q7) temporal precedence of mediator over outcome; and (Q8) control for confounding variables.

For Q3, sample size adequacy was evaluated pragmatically in light of the previous methodological literature on mediation analysis. In particular, Fritz and MacKinnon [46] showed that the required sample size in mediation models varies substantially depending on effect sizes and the statistical approach employed, with the median required sample size across simulated conditions being 187 participants (interquartile range: 107–352). Accordingly, sample size adequacy was interpreted in relative rather than absolute terms.

For Q5, “appropriate statistical methods” referred specifically to the use of established analytical procedures for mediation testing (e.g., regression-based approaches, structural equation modeling, bootstrap methods, or path analysis), rather than to the adequacy of the study design for causal mediation inference. Aspects related to temporal ordering and confounding control were instead evaluated separately through criteria Q6–Q8.

Methodological appraisal of the included studies was independently performed by three reviewers (F.C., L.C., and M.T.), who rated each item as present (1) or absent (0). Discrepancies were resolved through discussion with two additional reviewers (V.T. and C.F.) until consensus was reached.

### 2.6. Data Synthesis and Analysis

We initially summarized the characteristics of all eligible mediation studies. In the Results Section, studies were first classified by outcome (i.e., depression and pain intensity) and subsequently grouped by mediator (e.g., pain catastrophizing, helplessness, magnification). Results from cross-sectional and longitudinal studies were also explicitly distinguished throughout the synthesis to facilitate interpretation of the mediation findings according to study design and temporal assumptions. For each mediator, we conducted meta-analyses of the indirect, direct, and total effect estimates. A minimum of three eligible studies was set as the threshold for performing a meta-analytic synthesis, in line with common methodological practice to ensure adequate stability of pooled estimates.

When effect sizes and their corresponding standard errors were available, pooled indirect, direct, and total effects were estimated using random-effects models with the DerSimonian–Laird estimator (method = “DL”), implemented in the rma() function of the metafor package [47].

Statistical heterogeneity was assessed using the I^2^ statistic and the χ^2^ (Q) test. The I^2^ statistic quantifies the proportion of total variability across studies due to heterogeneity rather than chance, indicating the extent to which study findings were consistent or meaningfully different from one another. Given the small number of studies included in each meta-analysis and the clinical and methodological heterogeneity across studies (e.g., different CP populations, outcome measures, and mediation models), random-effects models were used throughout to account for between-study variability. The I^2^ statistic was therefore used to quantify and describe statistical heterogeneity rather than to determine model selection.

Given the limited number of studies included in each meta-analysis, publication bias was not formally assessed. Funnel plots were generated for exploratory purposes only and are provided in the Supplementary Materials.

A sensitivity analysis was also conducted using the leave-one-out function to assess the influence of individual studies on pooled estimates. This analysis allowed us to evaluate the robustness of the findings by determining whether the overall results were disproportionately influenced by any single study.

All analyses were conducted in R (version 4.3.0), primarily using functions from the metafor and meta packages. The R scripts and pooled estimates used for all analyses are provided as Supplementary Files S4–S7.

For those studies lacking sufficient quantitative data, we employed the vote-counting method to summarize the direction of the indirect effect for each mediator following the SWiM guideline [41]. Results were displayed using harvest plots, as recommended in the Cochrane Handbook [48]. For graphical display purposes only, appraisal scores were grouped into three ordinal categories (2–3, 4–5, and 6–7) to facilitate visualization in the harvest plots. These categories were not intended to represent validated thresholds of methodological quality.

Several studies evaluated more than one mediation model (e.g., cross-sectional and longitudinal analyses or models with different levels of covariate adjustment). In the narrative synthesis, the individual mediation model was considered the primary unit of analysis and was therefore retained separately in the vote counting and corresponding harvest plots to preserve these methodological distinctions. However, when interpreting the overall body of evidence, we explicitly considered whether multiple mediation models originated from the same study and sample. In particular, differences in study design or statistical adjustment across models from the same study were taken into account when interpreting the consistency and strength of the evidence, rather than treating these models as entirely independent findings.

## 3. Results

### 3.1. Study Selection

**Figure 2:**
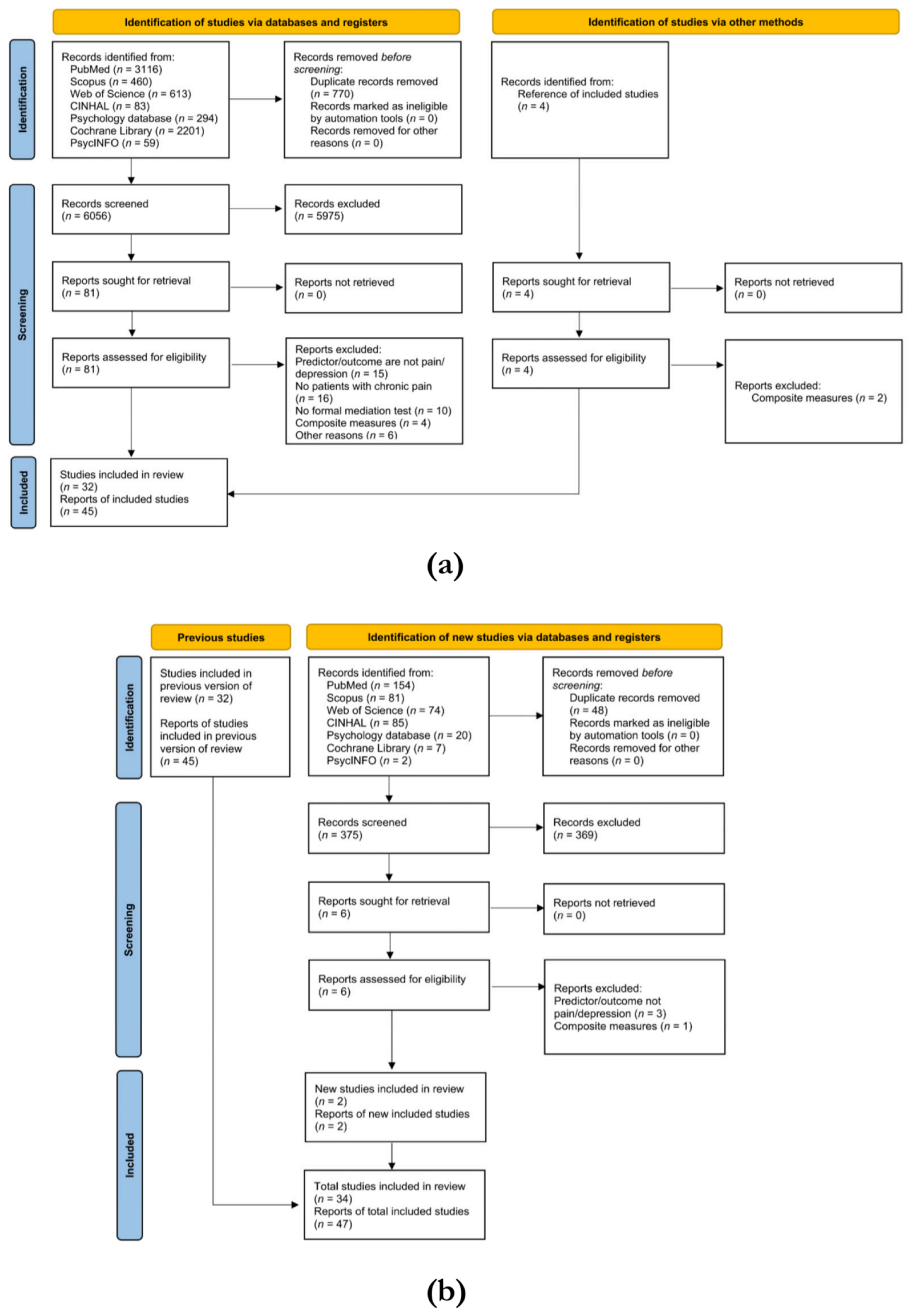
PRISMA 2020 flow diagrams of the study selection process. (**a**) Initial database search (May–July 2023). (**b**) Updated database search (May–June 2024).

The first search strategy identified a total of 6826 studies. After conducting the screening procedures, 81 studies were evaluated in full text to determine eligibility. An additional four studies were identified through hand-searching the reference lists of the eligible articles. Following full-text assessment, 32 studies (45 mediation models) met the inclusion criteria. The search update yielded 423 citations, 6 of which were assessed for eligibility. Following full-text assessment, two studies (two mediation models) met the inclusion criteria. Overall, this process resulted in a total of 34 relevant studies (47 models of mediation) to be included in the review (Figure 2).

### 3.2. Study Characteristics

The included studies were published between 2003 and 2024. They were conducted predominantly in the United States (nine studies, 26.4%), Spain (six studies, 17.7%), Australia (three studies, 8.8%), and China (three studies, 8.8%). The participants’ mean age ranged from 35.5 to 82.5 years; the percentage of females ranged from 10% to 100%.

Twenty-seven studies (79.4%; 36 models) had a cross-sectional design, while 8 studies (23.5%; 11 models) had a longitudinal one (although 34 studies were included overall, 1 study tested both a cross-sectional model and 2 longitudinal models and was therefore counted in both categories). Seven studies included patients with CP of any type [49,50,51,52,53,54,55]. Five studies included patients with fibromyalgia [56,57,58,59,60]. Five other studies included patients with spinal cord injury [37,61,62,63,64]; one of these studies also included patients with multiple sclerosis [62]. Four studies included patients with chronic low back pain [65,66,67,68]; one of these studies also included patients with chronic leg pain [65]. Three studies included patients with chronic musculoskeletal pain [69,70,71]. Three other studies included patients with injury-related chronic pain [72,73,74]. Two studies included patients with osteoarthritis [75,76]. Two studies included patients with rheumatoid arthritis [39,77]. Finally, single studies included patients with neck pain [78], chronic non-cancer pain [79], and interstitial cystitis/bladder pain syndrome [80].

Sample sizes for mediation analyses ranged from 112 to 1195.

Table 1 summarizes the study characteristics; Table 2 summarizes the variables, measurement tools, mediation analyses, and confounder adjustments of the included studies.

### 3.3. Measures of Pain Intensity

Eight studies [50,51,53,62,63,65,66,74] assessed pain intensity using an 11-point Numeric Rating Scale (NRS), while one study [37] used a 10-point NRS. Eight studies [57,58,60,61,68,70,77,80] used the Short Form of the McGill Pain Questionnaire (SF-MPQ) [81]. Four studies [54,55,71,79] used the “pain intensity” subscale of the Brief Pain Inventory (BPI) [82], while one study used only the BPI “average pain” item [55]. Three studies [67,72,78] used a 100 mm Visual Analog Scale (VAS), one study [52] used the “pain intensity” subscale of the Chronic Pain Grade (CPG) [83], and another study [49] used the pain intensity subscale of the West Haven–Yale Multidimensional Pain Inventory (WHYMPI) [84]. Other studies employed condition-specific measures: one study [76] of osteoarthritis patients used the pain subscale of the Western Ontario and McMaster University Osteoarthritis Index (WOMAC) [85]; one study [39] of patients with rheumatoid arthritis used the Rheumatoid Arthritis Pain Scale (RAPS) [86]; and one study of patients with spinal cord injury [64] used the “pain intensity” item of the International Spinal Cord Injury Pain Basic Data Set (ISCIPBDS) [87]. Further studies assessed pain intensity using composite measures: two studies [69,73] combined different NRS scores assessing different pain intensity levels (e.g., worst, least), while three studies [56,59,75] combined different pain scales or subscales in a single index.

For reasons of clarity, we are referring to “pain intensity” throughout the review, although “pain severity” is a term used in some of the included studies. These terms are often interchangeably used; however, there are various debates regarding the qualitative difference between these terms [35].

### 3.4. Measures of Depression

Depressive symptoms were most commonly assessed using the Hospital Anxiety and Depression Scale (HADS) [88] (eight studies) [39,57,58,59,60,67,70,72], followed by the Patient Health Questionnaire-9 (PHQ-9) [89] (six studies) [54,55,62,63,73,80], the “Depression” subscale of the Depression, Anxiety and Stress Scale (DASS) [90] (five studies) [50,51,53,77,79], the Center for Epidemiological Studies Depression Scale (CES-D) [91] (five studies) [49,52,64,66,75], and the Beck Depression Inventory (BDI) [92,93] (three studies) [65,68,74]. Less frequently used instruments included the Geriatric Depression Scale (GDS) [94] (two studies) [69,76], the 5-item Mental Health Index of the 36-Item Short-Form Health Survey (SF-36) [95] (one study) [37], the Profile of Mood States (POMS) [96] (one study) [61], the Zung Self-rating Depression Scale (ZSDS) [97] (one study) [78], and the 20-item Hopkins Symptom Checklist (HSCL-20) [98] (one study) [71]. Finally, one study [56] used a combined measure of depression, calculated as the sum of the standardized scores of the CES-D and the observer-rated Hamilton Rating Scale for Depression (HRSD) [99].

### 3.5. Methodological Appraisal of the Included Studies

The methodological appraisal was performed separately for each of the 47 mediation models included in the review. Appraisal scores ranged between 2 and 7 (out of 8), with both the median and mode equal to 5, indicating that most mediation models fulfilled approximately half of the appraisal criteria. Three mediation models obtained a score of 2 or 3, 26 of 4 or 5, and 18 of 6 or 7.

All studies clearly described the study objectives and reported the psychometric properties of the tools used to measure the mediator and outcome variables or their relevant reference. Most of the studies used appropriate statistical methods of mediation analysis (32/34, 94%) and cited a theoretical framework to explain the mechanism linking pain to depression or vice versa (28/34, 82%). Fewer studies used a sample that is adequate in terms of size or consistency with the research hypothesis (17/34, 50%) and controlled for possible confounding variables (20/34, 59%). Only two and six studies established, respectively, whether changes in the exposure preceded changes in the mediator [55,80] or whether changes in the mediator preceded changes in the outcome [55,72,73,74,76,80]. The appraisal scores for individual mediation models are presented in Table 3.

### 3.6. Results from the Mediation Studies: Narrative Synthesis and Meta-Analysis

#### 3.6.1. Mediators of the Pathway from Pain Intensity to Depressive Symptoms

Of the 34 included studies, 29, comprising a total of 13,587 participants, tested 43 models examining the relationship between pain intensity and depressive symptomatology [37,49,50,51,52,53,56,57,58,59,60,61,62,63,64,65,66,67,69,71,72,75,76,77,78,79,80].

Overall, 24 possible mediators on the hypothesized path from pain intensity to depression were identified. Quantitative synthesis was feasible only for two mediators (i.e., catastrophizing and helplessness), as the necessary data (e.g., standardized coefficients, standard errors) were unavailable for the remaining variables or because fewer than the minimum number of studies required for meta-analytic synthesis was available.

**Pain catastrophizing**

Pain catastrophizing was investigated by six studies—four with a cross-sectional design [52,60,65,79] and two with a longitudinal design [66,80]—yielding conflicting results. Three of these studies [52,60,79] found a mediating effect, while the remaining three studies [65,66,80] did not confirm this effect. Notably, all studies reporting a positive mediating effect were cross-sectional, whereas both longitudinal studies [66,80] failed to observe a significant mediation effect, suggesting a potential influence of study design on the observed associations. In addition, in two of the three studies with negative results [65,66], pain catastrophizing was measured using the “catastrophizing” subscale of the Kiel Pain Inventory, a different instrument from those used in the other studies more focused on evaluating the presence of thoughts in which the threatening nature of the pain is emphasized (e.g., “Surely there won’t be a serious illness behind this?”).

Meta-analysis. Four studies (*n* = 2419) [52,65,66,79] met the criteria to be included in the meta-analysis.

**Figure 3:**
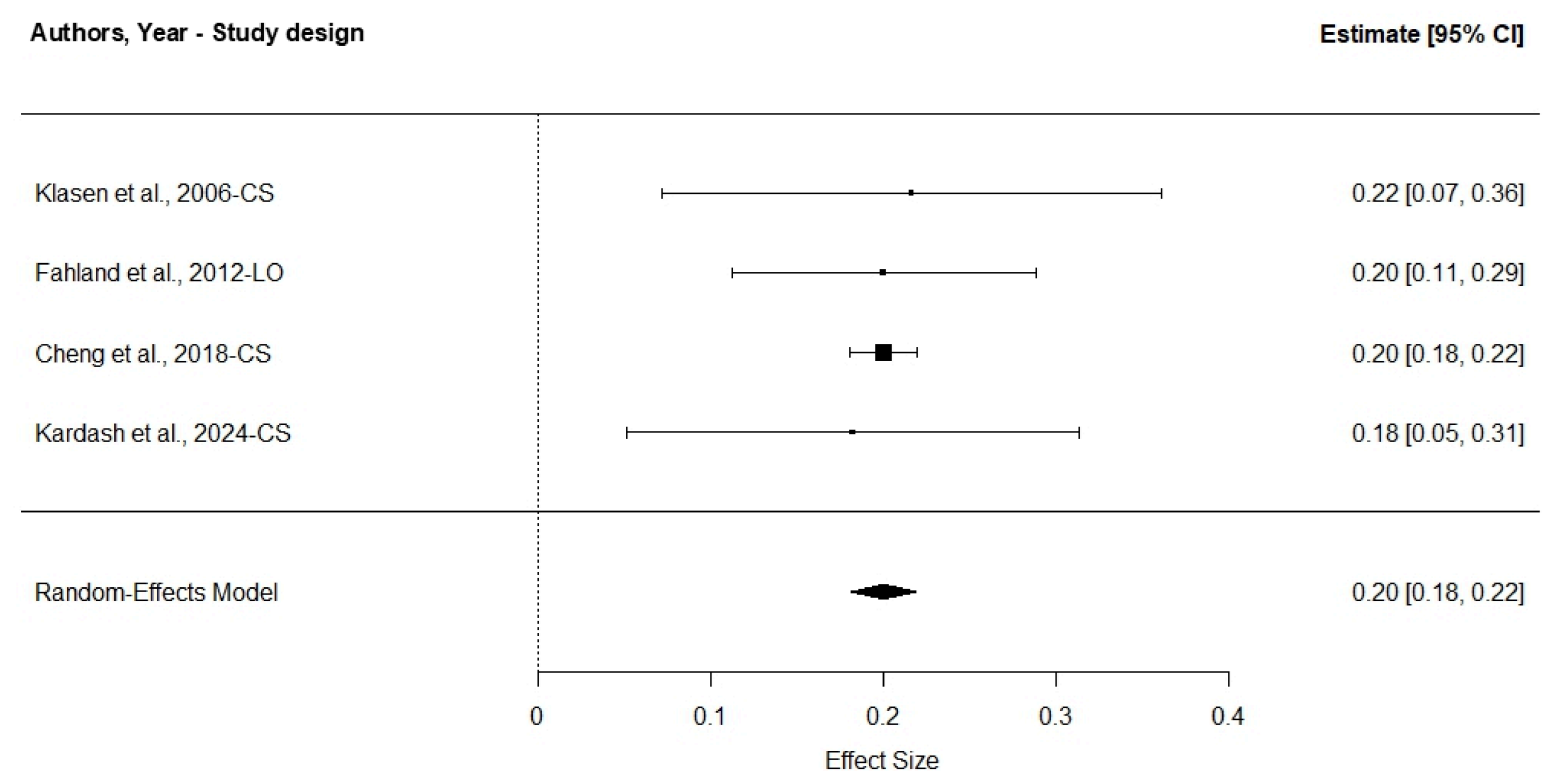
Meta-analysis of the total effect of pain intensity on depressive symptoms through pain catastrophizing, including the studies by Klasen et al. [65], Fahland et al. [66], Cheng et al. [52], and Kardash et al. [79].

The total effect of pain intensity on depressive symptomatology was significant and highly consistent across studies (random-effects model, β = 0.200, SE = 0.0096, 95% CI [0.181, 0.219], *p* < 0.0001), with no evidence of heterogeneity (I^2^ = 0%) (Figure 3).

**Figure 4:**
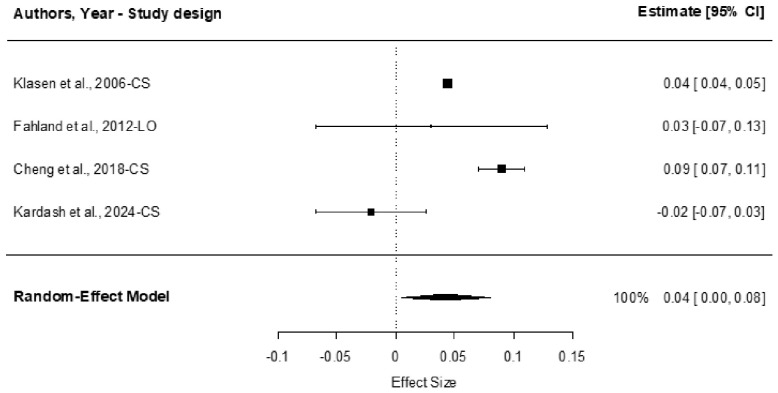
Meta-analysis of the direct effect of pain intensity on depressive symptoms after accounting for pain catastrophizing, including the studies by Klasen et al. [65], Fahland et al. [66], Cheng et al. [52], and Kardash et al. [79].

The direct effect, controlling for catastrophizing, was small but significant (random-effects model, β = 0.042, SE = 0.019, 95% CI [0.005, 0.080], *p* = 0.027), with high heterogeneity (I^2^ = 89.5%) (Figure 4).

**Figure 5:**
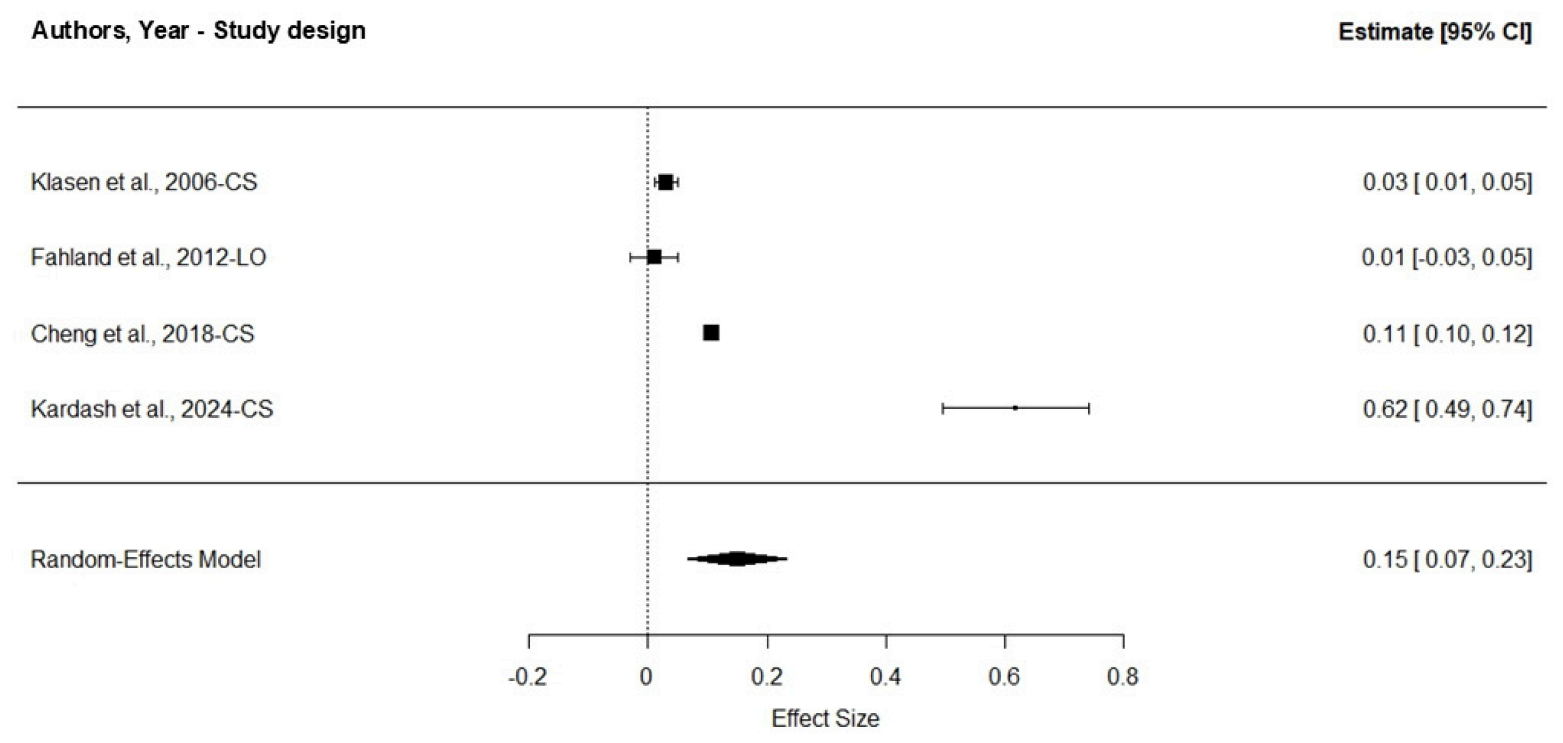
Meta-analysis of the indirect effect of pain intensity on depressive symptoms through pain catastrophizing, including the studies by Klasen et al. [65], Fahland et al. [66], Cheng et al. [52], and Kardash et al. [79].

The indirect effect of catastrophizing was significant (random-effects model, β = 0.150, SE = 0.043, 95% CI [0.066, 0.233], *p* = 0.0005), although substantial heterogeneity (I^2^ = 97.8%) suggests that this estimate should be interpreted cautiously (Figure 5).

Sensitivity analysis revealed that when the study by Kardash et al. [79] was excluded, the indirect effect dropped to β = 0.051 (*p* = 0.1251), losing statistical significance. This finding indicates that the pooled estimate was highly influenced by this study and should therefore be interpreted with caution.

Publication bias was not formally assessed due to the limited number of included studies; exploratory funnel plots are available in Supplementary File S8.

**Helplessness**

Helplessness was investigated by six studies (seven models), four with a cross-sectional design [50,56,65,71] and two with a longitudinal design [51,66], all of which found that helplessness mediates the relationship between pain intensity and depressive symptoms.

Meta-analysis. Four studies (*n* = 1341) [50,51,65,66] met the criteria to be included in the meta-analysis.

**Figure 6:**
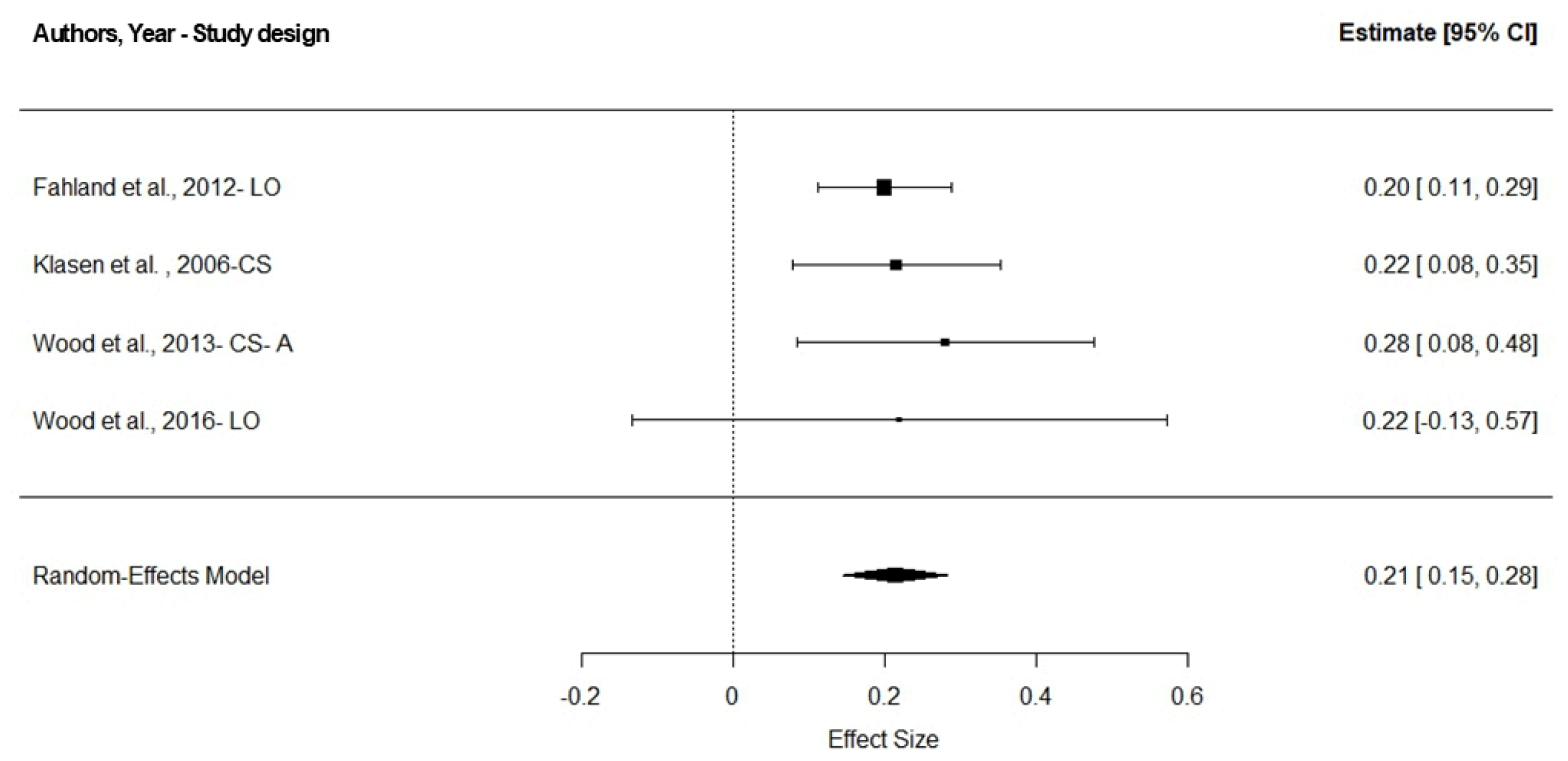
Meta-analysis of the total effect of pain intensity on depressive symptoms through helplessness, including the studies by Fahland et al. [66], Klasen et al. [65], Wood et al. [50], and Wood et al. [51].

The total effect of pain intensity on depressive symptomatology was significant (random-effects model, β = 0.214, SE = 0.035, 95% CI [0.146, 0.282], *p* < 0.0001), with no observed heterogeneity (I^2^ = 0%) (Figure 6). In the leave-one-out analyses, removal of each study in turn yielded pooled effects ranging from β = 0.205 to β = 0.236, all highly significant (*p* < 0.0001) and with overlapping confidence intervals. In all scenarios, heterogeneity remained null (I^2^ = 0%). These results indicate that no single study exerted undue influence on the overall effect, confirming the robustness and stability of the meta-analytic estimate. Given the limited number of studies, funnel plots were provided for exploratory purposes only (see Supplementary File S7) and should be interpreted with caution.

**Figure 7:**
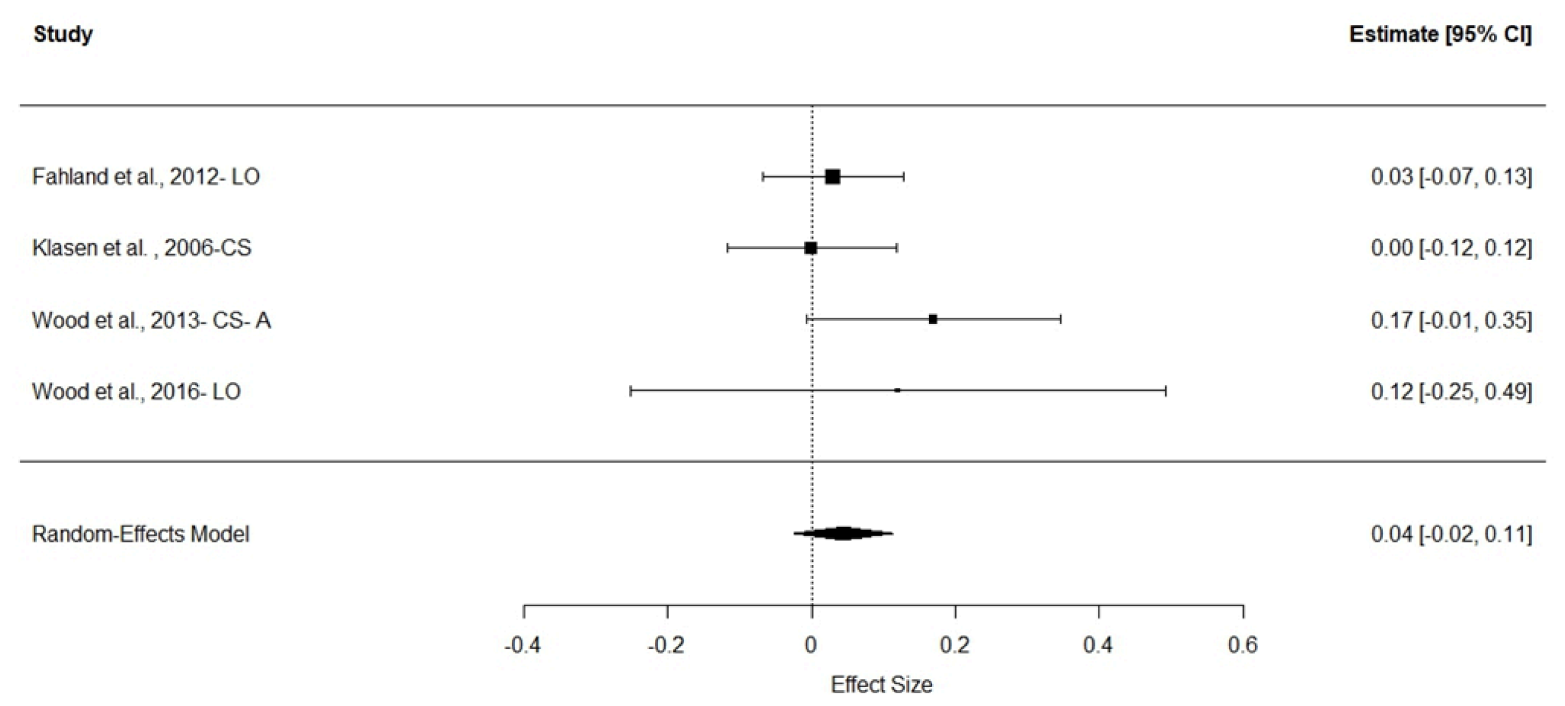
Meta-analysis of the direct effect of pain intensity on depressive symptoms after accounting for helplessness, including the studies by Fahland et al. [66], Klasen et al. [65], Wood et al. [50], and Wood et al. [51].

A random-effects meta-analysis was conducted on three studies reporting standardized direct effects. The direct effect was not significant (random-effects model, β = 0.044, SE = 0.034, 95% CI [−0.024, 0.112], *p* = 0.207), with no evidence of heterogeneity (I^2^ = 0%) (Figure 7).

**Figure 8:**
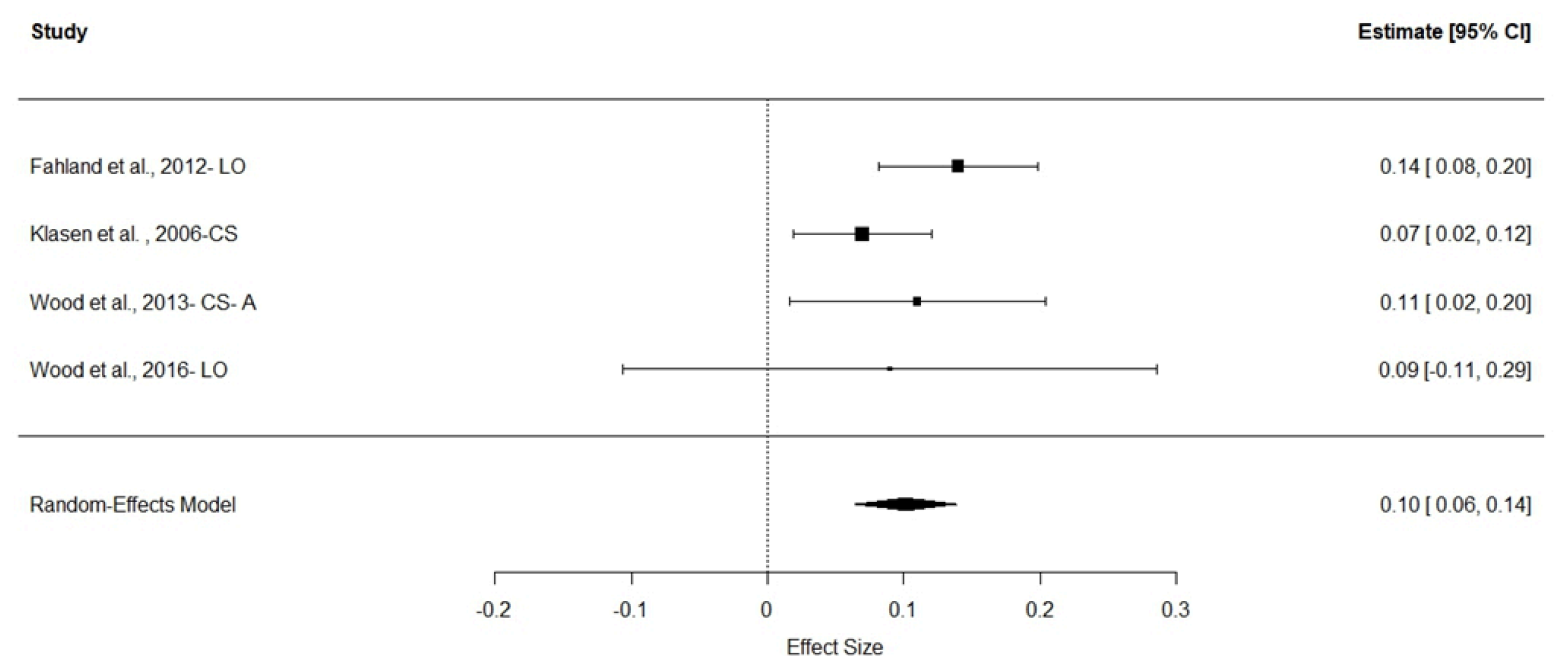
Meta-analysis of the indirect effect of pain intensity on depressive symptoms through helplessness, including the studies by Fahland et al. [66], Klasen et al. [65], Wood et al. [50], and Wood et al. [51].

The indirect effect was significant (random-effects model, β = 0.101, SE = 0.018, 95% CI [0.066, 0.137], *p* < 0.0001), with minimal heterogeneity (I^2^ = 4.65%) (Figure 8). A leave-one-out sensitivity analysis was performed to assess the influence of individual studies on the pooled random-effects estimate. The resulting pooled effects ranged from β = 0.080 to β = 0.130, all statistically significant (*p* < 0.001) and with overlapping confidence intervals. Heterogeneity remained minimal (I^2^ = 0–36%), and no single study meaningfully altered the magnitude or significance of the pooled effect.

As for pain catastrophizing, publication bias was not formally assessed due to the limited number of included studies; exploratory funnel plots are available in Supplementary File S9.

**Magnification**

Two studies (three models) investigated the role of magnification: one cross-sectional study [50] and one longitudinal study [51]. Both studies reported a significant mediating effect across all three models.

**Physical function**

Physical function was investigated by two studies (four models)—one cross-sectional study [75] and one longitudinal study [76]—both conducted on osteoarthritis samples but yielding conflicting results. Bookwala et al. [75] found that physical function does not mediate the relationship between pain intensity and depressive symptoms. The longitudinal study by Wang et al. [76] found that the mediation effect of physical function emerged in cross-sectional and initial longitudinal analyses, but it disappeared in a subsequent longitudinal model in which the depression score at the 6-month follow-up was adjusted by the baseline value.

**Social functioning**

Social functioning was investigated by a single cross-sectional study [75], which found that it does not mediate the relationship between pain intensity and depressive symptoms.

**Endurance**

Behavioral endurance in response to pain was investigated by a single cross-sectional study [65], which found that it fully mediates the relationship between the intensity of pain and depression.

**Pain interference**

Pain interference was investigated by five cross-sectional studies (nine models) [49,56,62,63,69]. Four studies (eight models) found that it mediates the relationship between pain intensity and depressive symptoms, while one study [56] did not confirm this effect. Of note, the only study that did not confirm pain interference as a mediator of the pain–depression relationship included multiple mediators (pain interference and helplessness) in its mediation analyses.

**Social support**

Social support was investigated by a single longitudinal study [72], which found no evidence of mediation, as pain did not predict social support (β = 0.06; *p* = 0.30).

**Sleep quality**

Sleep quality was investigated by three cross-sectional studies [57,58,78], all of which found that it mediates the relationship between pain intensity and depressive symptoms.

**Pain self-efficacy**

Pain self-efficacy was investigated by five studies. Four cross-sectional studies [57,58,59,61] found that it mediates the relationship between pain intensity and depressive symptoms; of note, one of these studies investigated the mediating role of different subdomains of pain self-efficacy and found that self-efficacy for physical function and for pain management completely mediated the relationship between pain and depression [59]. Conversely, the longitudinal study of Pjanic et al. [72] found no evidence of mediation for pain self-efficacy.

**Fear-avoidance beliefs**

Fear-avoidance beliefs were investigated by a single longitudinal study [66], which showed that they do not mediate the relationship between pain intensity and depressive symptoms.

**Rumination**

Rumination was investigated by a single longitudinal study [80], which found no evidence of mediation.

**Objective sleep efficiency**

One cross-sectional study [58] investigated the mediating role of objective sleep efficiency, showing that it fully mediates the relationship between pain intensity and depression.

**Sexual functioning**

One cross-sectional study [67] investigated the mediating role of sexual functioning, showing that it partially mediates the relationship between pain intensity and depressive symptoms.

**Positive and negative affects**

Positive and negative affects were investigated by a single cross-sectional study [59], which showed that they do not mediate the relationship between pain intensity and depressive symptoms.

**Participation**

Participation in daily activities was investigated by a single cross-sectional study testing three models of mediation [37]. The authors found that only participation restriction and satisfaction mediated the effects of pain intensity on depressive symptoms in individuals with tetraplegia and in those living for less than 10 years with an incomplete lesion (the first also in individuals with a complete lesion and living for less than 10 years with an incomplete injury). However, the study found no evidence of mediation for participation frequency.

**Pain acceptance**

Pain acceptance was investigated by two cross-sectional studies, yielding conflicting results. In one study [77], pain acceptance fully mediated the relationship between pain and depression, suggesting that higher levels of acceptance can significantly buffer the psychological impact of pain on depressive symptoms. Conversely, the second study [60] found no evidence of mediation, indicating that pain acceptance did not significantly influence the relationship between pain intensity and depression.

**Mindfulness**

Mindfulness was investigated by a single cross-sectional study [77], which found that only the ability of non-reacting (i.e., the tendency to non-react to one’s inner experiences as thoughts or feelings) fully mediates the relationship between pain intensity and depressive symptoms.

**Emotion regulation**

Emotion regulation was investigated by a single study with a longitudinal design [80], which found that it does not mediate the relationship between baseline pain and depression at one year.

**Exercise frequency**

Exercise frequency was investigated by a single cross-sectional study [78], which found that it does not mediate the relationship between the intensity of pain and depressive symptoms.

**Pain-related cognitions**

One cross-sectional study [71] investigated the mediating role of different pain-related beliefs concerning medical cure, medication, disability, emotion, solicitousness, control, and harm. Mediation analyses revealed that only beliefs about disability (i.e., the extent to which the patient believes that one is unable to function because of pain) fully mediate the relationship between pain intensity and depression.

**Quality of life**

Quality of life was investigated by a single cross-sectional study [64], which found that it fully mediates the relationship between pain intensity and depression.

**Behavioral coping**

One cross-sectional study [60] investigated the mediating role of behavioral coping, showing that it does not mediate the relationship between pain intensity and depressive symptoms.

**Cognitive fusion**

Cognitive fusion was investigated by a single cross-sectional study [53], which showed that it partially mediates the association between pain intensity and depressive symptoms.

Overall, mediation effects were more consistently observed in cross-sectional studies, whereas longitudinal findings were more heterogeneous and frequently non-significant, with the notable exceptions of helplessness and magnification.

**Figure 9:**
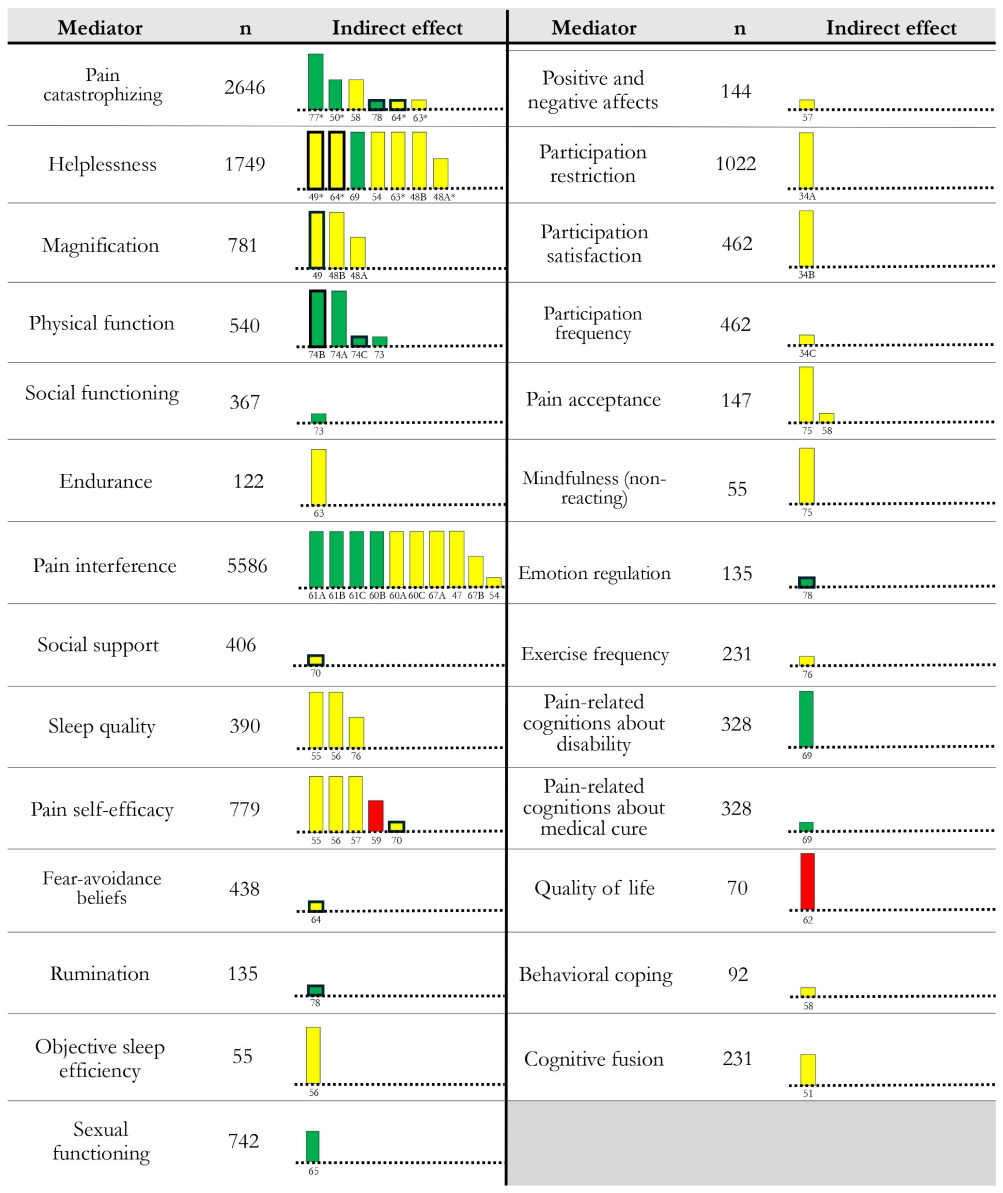
Harvest plot illustrating mediators between pain intensity and depressive symptoms. Bar height represents the strength of the mediation effect (tall = full mediation; medium = partial mediation; short = no mediation). Bar color indicates the methodological appraisal score (red = 2–3; yellow = 4–5; green = 6–7). Bar outline reflects study design (thin = cross-sectional; thick = longitudinal). Labels below each bar identify the corresponding study/model in the list above. An asterisk (*) near to the study indicates its inclusion in the meta-analysis.

Figure 9 presents a harvest plot summarizing the indirect effects identified by studies on mediators of the pain–depression relationship that were not included in the meta-analysis.

#### 3.6.2. Mediators of the Pathway from Depression to Pain

Of the 34 included studies, 9, comprising 1359 participants, tested nine models to evaluate the relationship between depressive symptomatology and pain intensity [39,54,55,68,70,71,73,74,80]. Twelve possible mediators of the hypothesized path from depressive symptoms to pain intensity have been identified. However, none of the proposed mediators in this direction could be meta-analytically examined due to insufficient or incomplete reporting of mediation estimates or because the available data were provided by fewer than the minimum number of studies required for meta-analytic synthesis.

**Pain catastrophizing**

Pain catastrophizing was investigated by three studies, one with a cross-sectional design [54] and two with a longitudinal design [73,80], all of which found that catastrophizing mediates the relationship between depressive symptoms and pain intensity.

**Illness perception**

Illness perception was investigated by a single cross-sectional study [39], which found that some of its dimensions, specifically “consequences”, “personal control”, and “emotional response”, partially mediate the relationship between depressive symptoms and pain intensity.

**Pain self-efficacy**

Pain self-efficacy was investigated by two studies, one with a cross-sectional design [68] and one with a longitudinal design [55]. Skidmore et al. [68] found that it mediates the relationship between depressive symptoms and pain, while the longitudinal study by Nephew et al. [55] did not confirm this effect.

**Emotion regulation**

Emotion regulation was investigated by a single study with a longitudinal design [80], which found that it does not mediate the relationship between baseline depression and pain at one year.

**Helplessness**

Helplessness was investigated by a single study with a longitudinal design [80], which found that it mediates the relationship between baseline depression and pain at one year.

**Magnification**

Magnification was investigated by a single study with a longitudinal design [80], which found that it does not mediate the relationship between baseline depression and pain at one year.

**Rumination**

Rumination was investigated by a single study with a longitudinal design [80], which found that it does not mediate the relationship between baseline depression and pain at one year.

**Pain-related cognitions**

One cross-sectional study [71] investigated the mediating role of different pain-related beliefs concerning medical cure, medication, disability, emotion, solicitousness, control, and harm. Mediation analyses revealed that only beliefs about harm (i.e., the degree to which the patient believes that pain means damage and thus activity should be restricted) and emotions (i.e., the degree to which the patient believes emotions affect pain) mediate the relationship between depression and pain intensity.

**Perceived stress**

Perceived stress was considered by a single study with a longitudinal design [55]. However, the authors found that baseline depression did not predict average pain nor pain severity; therefore, no mediation analyses were conducted.

**Sleep quality**

Sleep quality was considered by a single study with a longitudinal design [55]. However, the authors found that baseline depression did not predict average pain nor pain severity; therefore, no mediation analyses were conducted.

**Outcome expectancies**

Outcome expectancies were investigated by a single longitudinal study [74], which found that positive, but not negative, treatment outcome expectancies partially mediate the relationship between depressive symptoms and pain intensity.

**Central sensitization**

Central sensitization was investigated by a single cross-sectional study [70], which found that it fully mediates the relationship between depressive symptoms and pain intensity.

Overall, evidence from longitudinal studies investigating mediators of the depression–pain relationship was mixed. Pain catastrophizing and helplessness showed significant mediation effects across longitudinal analyses, whereas other mediators, including pain self-efficacy, emotion regulation, magnification, and rumination, were not supported. However, most mediators were investigated by single studies only, limiting comparisons across study designs and the possibility of identifying consistent longitudinal patterns.

**Figure 10:**
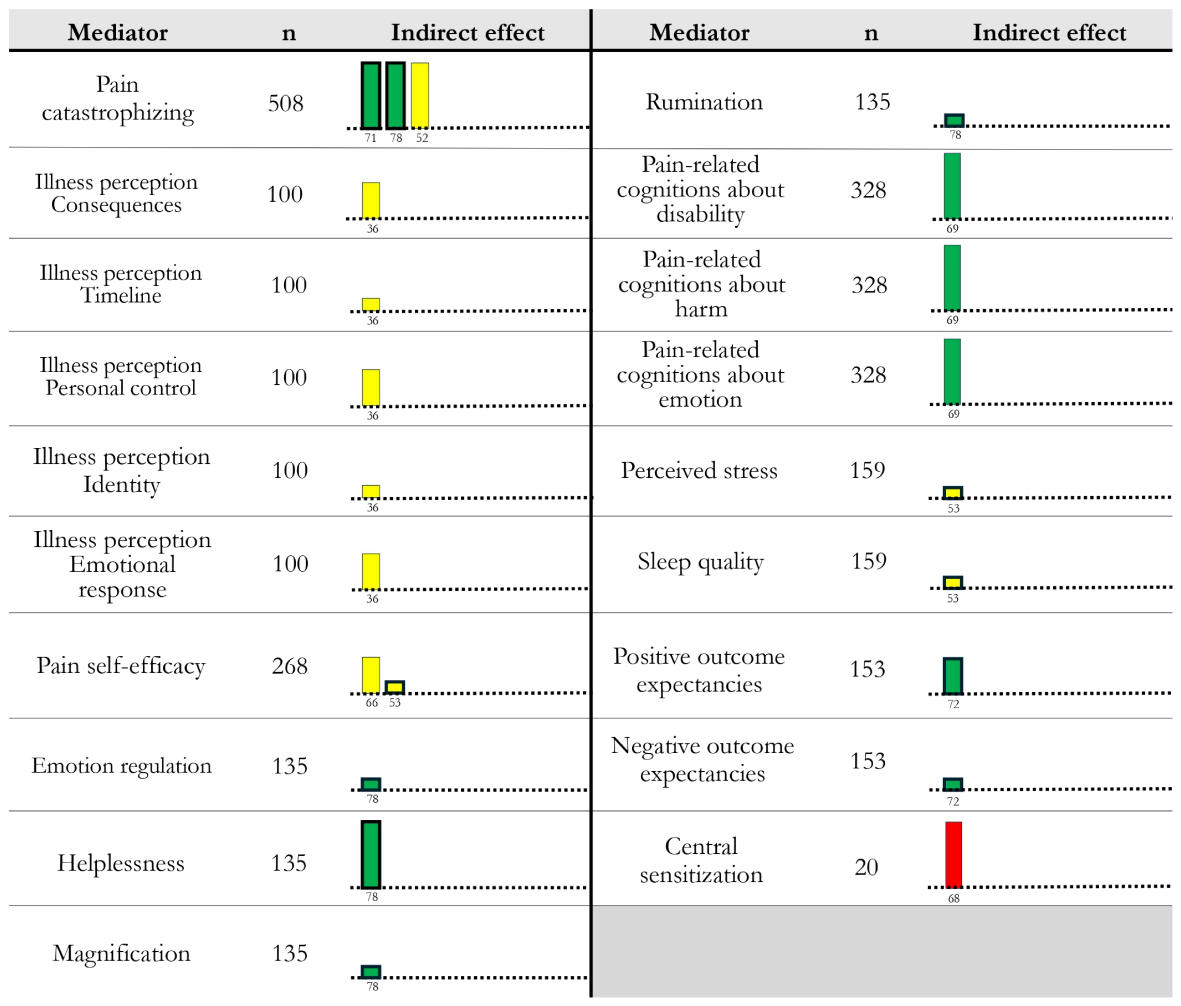
Harvest plot illustrating mediators between depressive symptoms and pain intensity. Bar height represents the strength of the mediation effect (tall = full mediation; medium = partial mediation; short = no mediation). Bar color indicates the methodological appraisal score (red = 2–3; yellow = 4–5; green = 6–7). Bar outline reflects study design (thin = cross-sectional; thick = longitudinal). Labels below each bar identify the corresponding study/model in the list above.

Figure 10 presents a harvest plot summarizing the indirect effects identified by the studies on mediators of the depression–pain relationship not included in the meta-analysis.

## 4. Discussion

Pain intensity and depressive symptoms frequently co-occur in individuals with CP [10,13,15], indicating a complex interplay in which each symptom domain may exacerbate the other, leading to greater disability, reduced quality of life, poorer treatment response, and higher healthcare and social costs [17,18]. While this bidirectional relationship is well established, the mechanisms through which pain and depressive symptoms influence one another have remained insufficiently synthesized. This systematic review, complemented by quantitative synthesis where feasible, aimed to address this gap by identifying and critically appraising candidate mediators of the pain–depression relationship.

Our meta-analysis identified pain catastrophizing and helplessness as the only candidate mediators of the pain-to-depression relationship for which quantitative synthesis was feasible. For pain catastrophizing, however, the pooled estimate should be interpreted cautiously because it was characterized by substantial heterogeneity and was strongly influenced by a single study. Moreover, important differences emerged between cross-sectional and longitudinal findings: while most cross-sectional studies supported an indirect effect of pain catastrophizing, neither of the two longitudinal studies detected a significant effect. Accordingly, the current evidence for pain catastrophizing should be regarded as preliminary and hypothesis-generating rather than as robust pooled evidence. In contrast, helplessness showed more consistent evidence across study designs and remained robust across sensitivity analyses, suggesting that perceived lack of control may represent a promising candidate mediator of the pain-to-depression relationship. These findings are consistent with theoretical models such as the learned helplessness theory [100] and the fear-avoidance model [101], both of which emphasize the role of negative cognitive appraisals of pain in fostering depressive symptoms. The stronger evidence for helplessness also suggests the importance of perceived controllability in the psychological adjustment to pain and supports clinical approaches aimed at enhancing self-efficacy and coping abilities.

One possible explanation for the more consistent findings observed for helplessness is its greater conceptual specificity. Whereas pain catastrophizing is conceptualized as a broader cognitive–affective construct encompassing rumination, magnification, and feelings of helplessness [102], helplessness specifically reflects a more focused cognitive schema centered on the perceived inability to control pain and its consequences. This narrower focus may make helplessness a more stable predictor of depressive symptomatology, particularly in longitudinal models where evidence for pain catastrophizing appeared substantially weaker. On the other hand, the apparent advantage of helplessness should be interpreted cautiously due to the potential conceptual overlap between helplessness-related cognitions and the cognitive–affective components of depressive symptom measures. Feelings of uncontrollability, pessimism, and perceived inability to cope may partially overlap with depressive symptomatology itself, thereby inflating associations between helplessness and depression outcomes. Consequently, part of the mediation effect may reflect shared construct content rather than a fully distinct mechanistic pathway. Moreover, two of the three studies reporting null findings for pain catastrophizing used the catastrophizing subscale of the Kiel Pain Inventory, which primarily emphasizes the threatening nature of pain (e.g., “Surely there won’t be a serious illness behind this?”) and may therefore capture magnification-related aspects more than helplessness-related cognitions. Nevertheless, the consistency of findings across longitudinal studies and sensitivity analyses suggests that helplessness may represent a clinically meaningful process within the pain–depression relationship, although future studies should further disentangle conceptual overlap from temporal and causal mechanisms.

Several other variables also emerged as candidate mediators of the pain–depression relationship, although current evidence remains limited. Among these, pain self-efficacy, sleep quality, pain interference, and physical function received the most consistent support across the included studies.

Pain self-efficacy was supported as a candidate mediator of the relationship between pain and depression in four out of five studies, suggesting that individuals who believe in their ability to function despite pain may be less likely to develop depressive symptoms. These findings are also consistent with the more robust evidence for the mediating role of helplessness. While helplessness reflects a perception of uncontrollability and passivity, pain self-efficacy represents its conceptual opposite, emphasizing perceived control and personal agency. This convergence further supports the central role of perceived control in emotional adjustment to CP, consistent with the transactional model of stress [103], suggesting that adaptive coping and self-efficacy may buffer the emotional impact of pain. Similarly, classical cognitive-behavioral models of pain and depression emphasize that negative beliefs about one’s ability to cope with pain or its consequences foster emotional distress and behavioral disengagement, creating a sequence of avoidance and mood deterioration. Conversely, enhancing self-efficacy can interrupt the cycle, promoting resilience by encouraging adaptive coping strategies and goal-directed behavior [36]. Sleep quality also emerged as a consistently supported candidate mediator in all three studies included in the review. This finding reinforces conclusions from previous systematic reviews and meta-analyses, which identified sleep disturbances as a relevant contributor in the pain–depression relationship [44]. Recent evidence has also clarified the mechanisms through which poor sleep may mediate the pain–depression relationship. For example, it has been shown that sleep disturbances may increase stress and negative affect and impair emotion regulation, thereby intensifying the emotional burden of pain and fostering depressive symptoms [104,105,106]. However, findings on pain self-efficacy and sleep quality should be interpreted cautiously, as the available evidence is based on a limited number of predominantly cross-sectional studies, with several limitations identified in the methodological appraisal. Given that several mediation effects identified in cross-sectional studies were not consistently replicated longitudinally, adequately powered prospective studies are needed to clarify the temporal role of these candidate mediators.

Pain interference (i.e., the extent to which pain disrupts daily activities) was another frequently supported candidate mediator, with four out of five studies reporting significant effects in our review. In contrast, physical function, which typically refers to an individual’s objective capacity to perform physical tasks, showed weaker evidence, with only two out of four models supporting its possible mediation role. This discrepancy in the mediating role of pain interference and physical functioning may be explained by the “Activity restriction” [107] and “Illness intrusiveness” [108] models, which posit that the interference of pain in meaningful activities and the resulting loss of positive experiences contributes more to emotional distress than objective functional limitations. As such, pain interference may be more tightly linked to depressive outcomes, which are often shaped by perceived losses and reduced participation in rewarding activities [109]. Notably, in the study of Wang et al. [76], the two positive findings derived from one cross-sectional and one longitudinal model became non-significant after adjusting for additional covariates in a second longitudinal model. This suggests that the evidence supporting the mediating role of physical function remains limited and needs further research.

Although most studies have examined how pain intensity contributes to depressive symptoms, fewer have explored the reverse pathway—from depression to pain. Among the available studies, pain catastrophizing was the most frequently investigated candidate mediator, with all three studies reporting significant indirect effects. Unlike the pain-to-depression pathway, longitudinal studies consistently supported this association, although the evidence remains limited. One possible explanation is that depressive symptoms may promote catastrophic interpretations of pain, thereby amplifying pain perception [102].

In our literature search, the only study that formally tested sleep as a potential mediator of the relationship between depression and pain found no evidence of mediation [55]. In contrast, a meta-analysis of cohort and case–control studies on depression, sleep disturbance, and CP reported a significant partial mediation effect of sleep disturbance on the relationship between depression and CP [44]. These contrasting findings underscore the need for further mediation studies examining the role of sleep in the depression-to-pain relationship.

Overall, the depression-to-pain pathway remains underexplored. Although several psychological factors have been proposed as candidate mediators, the limited number of available studies precludes firm conclusions and meaningful comparisons between cross-sectional and longitudinal evidence.

Surprisingly, none of the included mediation studies investigated biological mechanisms despite growing evidence suggesting that the interplay between pain and depression may involve shared neurobiological processes, including neuroinflammation, neurotransmitter dysregulation, and alterations in the endogenous opioid system [110,111,112]. Future mediation studies integrating biological and psychological factors may provide a more comprehensive understanding of pain–depression comorbidity.

### 4.1. Implications for Clinical Practice

Our findings have significant implications for research and clinical practice.

The systematic review and meta-analysis identified several candidate mediators in the pain–depression relationship, including helplessness, pain self-efficacy, sleep quality, and pain interference, all of which represent promising treatment targets. In particular, interventions should address control-related beliefs by fostering self-efficacy, for example through cognitive-behavioral therapy (CBT), which has shown effectiveness in individuals with CP in reducing both pain intensity and depressive symptoms or emotional distress [113,114,115]. Similarly, interventions targeting sleep disturbances, particularly CBT for insomnia (CBT-I), may help reduce the emotional burden of pain, although evidence regarding long-term effects remains limited [116,117]. Finally, reducing pain interference by promoting engagement in valued activities may also yield better emotional outcomes than focusing solely on improving physical function. CBT has proven to be an effective approach in reducing pain interference, likely through its focus on activity pacing, behavioral activation, and cognitive restructuring [113]. Acceptance and Commitment Therapy (ACT) has also shown particularly promising results. Unlike CBT, ACT specifically supports sustained engagement in valued activities despite persistent pain. Meta-analytic evidence shows that ACT produces moderate effects on pain interference post-treatment and even larger effects at follow-up, supporting its efficacy not only in the short term but also over time [118,119].

### 4.2. Implications for Research

Beyond identifying candidate mediators, this review also provides important insights into methodological priorities for future mediation research, derived from the critical appraisal and synthesis of the available evidence. Comparison of cross-sectional and longitudinal evidence suggests that some mediation effects may weaken or disappear when temporal ordering and baseline outcome levels are appropriately controlled, as observed for pain catastrophizing and, to a lesser extent, physical function. These findings reinforce the need for adequately powered longitudinal mediation studies to identify temporally robust candidate mediators linking pain and depression. This interpretation is consistent with methodological evidence showing that cross-sectional mediation analyses may not adequately capture longitudinal processes and can lead to substantially different conclusions compared to longitudinal models, particularly when temporal dynamics are explicitly modeled [120]. Moreover, cross-sectional estimates of mediation effects have been shown to produce biased or misleading inferences about indirect and direct effects, even under conditions where mediation is assumed to be complete, further highlighting the limitations of relying on cross-sectional data to infer causal pathways [121].

Future studies should also ensure adequate control for relevant confounders, including demographic characteristics, comorbidities, and baseline outcome levels, to reduce bias in mediation estimates and improve the interpretation of indirect effects. Without adequate adjustment for confounders and baseline levels of pain and/or depression, it becomes difficult to determine whether some observed indirect effects reflected true mediational processes or shared variance among closely related constructs.

Future studies should prioritize three-wave longitudinal designs to test whether changes in a mediator precede changes in the outcome while accounting for prior levels of both. Such designs are especially needed for factors already examined in multiple cross-sectional studies (e.g., pain catastrophizing, self-efficacy, sleep quality). Controlling for relevant covariates is also critical, as neglecting them may inflate mediation effects or misattribute causality. For example, in the study by Wang et al. (2015), functional disability mediated the relationship between pain and depressive symptoms only when depression at follow-up was not adjusted for baseline levels [76].

Adequate sample size should be determined through a priori power analyses based on the expected magnitude of indirect effects and the planned analytical approach [46]. Improving methodological rigor in this field also requires greater standardization in both measurement and reporting practices. The use of validated and standardized instruments for predictors, mediators, and outcomes, together with complete reporting of mediation parameters (e.g., standardized β coefficients and standard errors for each path and indirect effect), would substantially enhance comparability across studies and facilitate future quantitative syntheses. In addition, future studies should increasingly adopt theory-driven multiple mediation models to account for the complex interrelationships among candidate mediators. Psychological, behavioral, and functional factors are likely to influence one another rather than acting as independent mechanisms, and associations identified in simple mediation models—particularly those with small effect sizes—may not remain significant once additional mediators are considered [122].

Finally, future studies should consistently assess pain duration to ensure accurate identification of chronic pain populations and improve comparability across studies.

Key recommendations for future mediation research in the field of pain–depression comorbidity are summarized in Table 4.

### 4.3. Limitations

The present review has limitations that arise both from the characteristics of the available primary evidence and from methodological aspects of the review itself. Importantly, these two levels are closely intertwined, as several limitations of the available evidence directly affected the scope and strength of the present synthesis.

#### 4.3.1. Limitations of the Available Primary Evidence

The first and most important limitation concerns the available primary evidence. Most included studies employed a cross-sectional design (27/34, 79.4%), which substantially limits conclusions regarding temporal mechanisms and causal pathways. This limitation is particularly relevant because several mediation effects supported by cross-sectional studies were not consistently replicated in longitudinal analyses, suggesting that some proposed mediators may not represent temporally robust mechanisms. Moreover, methodological research has shown that cross-sectional mediation analyses may produce substantially different conclusions from longitudinal models and may yield biased estimates of indirect effects when temporal ordering cannot be established [46,122]. Consequently, although the present meta-analyses provide a quantitative synthesis of the available mediation effects, they should not be interpreted as establishing causal mediation pathways. Rather, they summarize observational evidence on candidate mediators, whose temporal validity and causal role require confirmation in adequately designed longitudinal studies and, where appropriate, experimental research.

Another important limitation of the primary literature concerns the insufficient control of potentially relevant confounders, including demographic characteristics, comorbidities, and baseline levels of pain and/or depressive symptoms. As illustrated by some longitudinal studies included in this review, mediation effects may weaken or disappear after appropriate adjustment for baseline outcome levels and other confounders, making it difficult to distinguish true mediation processes from shared variance among related constructs. In addition, several primary studies relied on relatively small samples, which may have reduced statistical power and contributed to unstable mediation estimates and inconsistencies across findings.

A further limitation of the available evidence concerns the incomplete reporting of mediation analyses. Many studies did not report the statistical information (e.g., standardized or unstandardized coefficients and standard errors for all mediation paths) required to estimate pooled effects. Clear and standardized reporting of mediation results is therefore essential to facilitate future meta-analytic work and improve the comparability of findings across studies. This limitation directly affected the present review by restricting the number of studies that could be quantitatively synthesized.

#### 4.3.2. Limitations of the Present Review

The review itself also has several limitations. Most importantly, the quantitative synthesis was based predominantly on cross-sectional evidence, reflecting the composition of the available primary literature. Consequently, although the present meta-analyses provide a quantitative synthesis of the available mediation effects, the pooled estimates should be regarded as preliminary and interpreted with caution rather than as evidence of causal mediation pathways. Instead, they summarize the available observational evidence on candidate mediators, whose temporal validity and causal role require confirmation in adequately designed longitudinal studies and, where appropriate, experimental research. Furthermore, only a small number of studies provided sufficient statistical information to be included in the quantitative synthesis. Despite efforts to contact corresponding authors, the necessary data were frequently unavailable, precluding subgroup analyses based on pain condition, study design, or sample characteristics. Similarly, sensitivity analyses, particularly leave-one-out procedures, become increasingly influenced by individual studies when only a few studies are available.

A further limitation is that we included only studies that conducted formal mediation analyses, typically using individual-level data and regression-based approaches. By doing so, we may have excluded studies that could have been considered if alternative methodological strategies had been adopted. For example, Karimi et al. [44] conducted a meta-analysis of longitudinal studies examining links between depression, sleep, and chronic pain using two-stage structural equation modeling (TSSEM) to combine correlation matrices from studies reporting different association measures (e.g., odds ratios, incidence rate ratios, Cohen’s *d*, Hedges’ *g*, or Pearson’s *r*). This illustrates how alternative methodological frameworks may incorporate a broader range of studies and provide complementary evidence.

Similarly, other studies were not included because they were not formally classifiable as mediation studies, although they provided valuable insights into potential mechanisms linking pain and depression. For example, network analysis estimates partial correlations among variables and can identify key connecting nodes within complex symptom networks [123]. A recent network analysis [124] identified pain catastrophizing as a central node linking pain, anxiety, and depression. Although such approaches do not formally test mediation, they may provide complementary information on potential mechanisms and could usefully be integrated into future evidence syntheses.

As a further limitation, we considered CP as an overarching phenotype, without restricting the review to specific pain conditions or clinical subgroups. On the one hand, this represents a strength, as it allows the identification of potentially transdiagnostic mechanisms operating across different CP populations. On the other hand, it may obscure mechanisms that are specific to particular populations, such as older adults or individuals with specific medical conditions. Future reviews focusing on more homogeneous pain populations may therefore complement and refine the present findings.

The methodological appraisal was based on an adapted version of the instrument proposed by Mansell et al., which evaluates methodological aspects specific to mediation analyses rather than providing a comprehensive assessment of risk of bias. Furthermore, no validated framework or standardized thresholds are currently available for appraising mediation studies, particularly those using observational designs. Future methodological research should therefore focus on developing and validating appraisal and risk-of-bias tools specifically tailored to mediation analyses across different study designs.

Finally, the review was restricted to studies published in English, Italian, and German; consequently, the possibility of language bias cannot be completely excluded.

## 5. Conclusions

This systematic review and meta-analysis synthesized current evidence on factors proposed to mediate the relationship between pain intensity and depressive symptoms in adults with CP. Findings suggest that control-related cognitions, sleep quality, and pain interference are among the most consistently supported candidate mediators. In particular, helplessness emerged as the most consistently supported candidate mediator of the association between pain intensity and depressive symptoms, underscoring the importance of perceived controllability in psychological adjustment to CP. Other factors, including pain self-efficacy, sleep quality, and pain interference, also showed preliminary evidence of mediation, identifying potentially promising intervention targets. However, evidence from longitudinal studies was generally weaker and less consistent than findings from cross-sectional studies, particularly for pain catastrophizing and physical function, underscoring the need for caution when interpreting mediation effects derived from cross-sectional data alone. Evidence on the reverse pathway, from depression to pain, remains limited, with pain catastrophizing representing the most consistently supported candidate mediator identified to date. Importantly, beyond identifying candidate mediators, the key finding of this review is the substantial gap between the large number of cross-sectional mediation studies and the limited availability of rigorous longitudinal evidence. Addressing this gap should be considered a priority for the field. Future research should prioritize theoretically grounded three-wave longitudinal designs, a priori power analyses, standardized measures and reporting practices, and appropriate control of confounding variables. Such methodological advances are essential to establish the temporal validity of proposed mediators and to strengthen causal inference regarding proposed mediators and inform the development of interventions targeting potentially modifiable mechanisms underlying the reciprocal relationship between pain and depression.

## Figures and Tables

**Table 1:** Characteristics of the included studies.

First Authors (Year)	Country	CP Syndrome	Study Design	Sample Size		Participants’ Characteristics	
				Baseline	Follow-Up	Mean Age (SD)	% of Females
Bookwala (2003)	US	OA	CS	367	NA	67.9 (9.7)	63.9
Klasen (2006)	DE	Chronic back/leg pain	CS	122	NA	M: 45.58 (11.10); F: 44.78 (11.68)	53.3
Cannella (2007)	US	CP (any type)	CS	141	NA	46 (8.2)	60
Palomino (2007)	US	FM	CS	80	NA	52.5 (13.17)	95
Mirò (2011)	ES	FM	CS	104	NA	46.39 (7.60)	100
Fahland (2012)	DE	Chronic back pain	LO	438	438	52.9 (NR)	59.6
Craig (2013)	AU	SCI-related CP	CS	70	NA	47.1 (12)	10
Wood (2013)—A	US	CP (any type)	CS	669	NA	71.8 (7.53)	57.1
Wood (2013)—B	US	CP (any type)	CS	98	NA	NR (NR)	NR
Diaz-Piedra (2014)	ES	FM	CS	55	NA	47.62 (7.64)	100
Lopez-Lopez (2014)—A	ES	Chronic MSK pain	CS	102	NA	82.5 (7.1)	84.3
Lopez-Lopez (2014)—B	ES	Chronic MSK pain	CS	106	NA	68.7 (8.1)	84.85
Molton (2014)—A	US	SCI-related CP	CS	90	NA	37 (NR)	96.7
Molton (2014)—B	US	SCI-related CP	CS	367	NA	54.9 (NR)	51.5
Molton (2014)—C	US	SCI-related CP	CS	64	NA	71 (NR)	51.6
Pjanic (2014)	CH	Injury-related CP	LO	406	274	43.24 (12.4)	19.3
Rezaei (2014)	IR	RA	CS	100	NA	45.46 (12.67)	72
Pakpour (2015)	IR	CLBP	CS	742	NA	M: 48.51 (13.46); F: 43.04 (11.17)	53
Peñacoba Puente (2015)	ES	FM	CS	144	NA	50 (11)	100
Skidmore (2015)	ENG	CLBP	CS	109	NA	42.6 (12)	68
Wang (2015)—A	CN	OA	CS	173	NA	62 (NR)	83
Wang (2015)—B	CN	OA	LO	173	136	62 (NR)	83
Wang (2015)—C	CN	OA	LO	173	136	62 (NR)	83
Wood (2016)	AU	CP (any type)	LO	141	112	73.92 (5.75)	63.1
Briest & Bethge (2017)	DE	Injury-related CP	LO	241	241	47.1 (9.8)	54.4
Müller (2017)—A	CH	SCI-related CP	CS	1022	NA	52 (NR)	28.5
Müller (2017)—B	CH	SCI-related CP	CS	462	NA	52 (NR)	28.5
Müller (2017)—C	CH	SCI-related CP	CS	462	NA	52 (NR)	28.5
Cheng (2018)	CN	CP (any type)	CS	664	NA	74.79 (7.2)	85.1
Lami (2018)	ES	FM	CS	92	NA	50.21 (8.15)	100
Carvalho (2019)	PT	CP (any type)	CS	231	NA	48.51 (10.89)	100
Costa (2019)	PT	RA	CS	55	NA	M: 52.82 (16.86); F: 55.93 (17.84)	80
Crawford (2019)—A	US/CA	Chronic interstitial cystitis/bladder pain	LO	135	135	52.57 (15.51)	100
Crawford (2019)—B	US/CA	Chronic interstitial cystitis/bladder pain	LO	135	135	52.57 (15.51)	100
Crawford (2019)—C	US/CA	Chronic interstitial cystitis/bladder pain	LO	135	135	52.57 (15.51)	100
Shigetoh (2019)	JP	Chronic MSK pain	CS	20	NA	67.5 (15.6)	60
Juan (2020)	CN	Chronic neck pain	CS	231	NA	48.9 (13.9)	71
Sanchez-Rodrıguez (2020)—A	ES	Chronic MSK pain	CS	328	NA	60.35 (10.17)	83
Sanchez-Rodrıguez (2020)—B	ES	Chronic MSK pain	CS	328	NA	60.35 (10.17)	83
Kim (2021)	US/ROK	CP (any type)	CS	132	NA	78.3 (6.68)	81.8
Li (2022)—A	US	SCI-related CP	CS	1990	NA	48.8 (15.2)	26.2
Li (2022)—B	US	SCI-related CP	CS	1062	NA	48.8 (15.2)	26.2
Li (2022)—C	US	SCI-related CP	CS	1584	NA	48.8 (15.2)	26.2
Nephew (2022)	US	CP (any type)	LO	159	141	IG: 50 (12.1); CG: 51 (12.4)	IG: 83; CG: 89
Paré (2023)	CA	Injury-related CP	LO	153	153	36.86 (10.33)	57
Kardash (2024)	AU	Non-cancer CP	CS	1195	NA	52.69 (14.27)	66.1
Williams (2024)	ZA	SCI-related CP	CS	70	NA	35.48 (9.34)	12.9

Note: For studies reporting multiple mediation models, letters (A, B, etc.) are used solely to distinguish the different mediation models analyzed within the same study. Abbreviations: AU, Australia; CA, Canada; CG, Control Group; CLBP, Chronic Low Back Pain; CN, China; CP, Chronic Pain; CS, Cross-Sectional; DE, Germany; ES, Spain; F, Females; FM, Fibromyalgia; IG, Intervention Group; IR, Iran; JP, Japan; LO, Longitudinal; M: Males; MSK, Musculoskeletal; NA, Not Applicable; NR, Not Reported; OA, Osteoarthritis; PT, Portugal; RA, Rheumatoid Arthritis; ROK, Republic of Korea; SCI, Spinal Cord Injury; US, United States; ZA, South Africa.

**Table 2:** Summary of the variables, measurement tools, mediation analyses, and confounder adjustments in the included studies.

First Authors (Year)	Exposure (Measure)	Mediator (Measure)	Outcome (Measure)	Mediation Approach	Significance Test	Confounder Adjustments
Bookwala (2003)	Pain (PGC-P and AIMS-2-P combined)	Physical function (AIMS-2-PF); Social functioning (MAI-A)	Depression (CES-D)	SEM	Sobel	Yes
Klasen (2006)	Pain (11-point NRS)	Pain catastrophizing (KPI-C); Help-/Hopelessness (KPI-H); Endurance (KPI-B)	Depression (BDI)	Baron and Kenny	Sobel	Yes
Cannella (2007)	Pain (WHYMPI-P)	Pain Interference (WHYMPI-I)	Depression (CES-D)	SEM	NR	No
Palomino (2007)	Pain (Combined score from TP count, MPQ-P, and FIQ-P)	Helplessness (RAI Helplessness subscale); Pain interference (WHYMPI-I)	Depression (Combined depression score derived from CES-D and SIGHD)	Baron and Kenny	Sobel	Yes
Mirò (2011)	Pain (MPQ-SF)	Sleep quality (PSQI); Chronic Pain Self-Efficacy (CPSS)	Depression (HADS)	Baron and Kenny	Sobel	Yes
Fahland (2012)	Pain (11-point NRS)	Pain catastrophizing (KPI-C); Help-/Hopelessness (KPI-H); Fear-avoidance beliefs (FABQ)	Depression (CES-D)	Preacher and Hayes	Bootstrapped CIs	No
Craig (2013)	Pain (MPQ-SF)	Self-efficacy (MSES)	Depression (POMS)	SEM	NR	No
Wood (2013)—A	Pain (11-point NRS)	Helplessness (PRSS-C Helplessness subscale); Magnification (PRSS-C Magnification subscale)	Depression (DASS-21 Depression subscale)	Baron and Kenny	Sobel	Yes
Wood (2013)—B	Pain (11-point NRS)	Helplessness (PRSS-C Helplessness subscale); Magnification (PRSS-C Magnification subscale)	Depression (DASS-21 Depression subscale)	Baron and Kenny	Sobel	No
Diaz-Piedra (2014)	Pain (MPQ-SF)	Objective sleep efficiency (Polysomnography); Sleep quality (PSQI); Chronic Pain Self-Efficacy (CPSS)	Depression (HADS)	Baron and Kenny	Sobel	No
Lopez-Lopez (2014)—A	Pain (current, worst, least, and average pain combined into an overall 11-point NRS)	Pain interference (ARS)	Depression (GDS)	Preacher and Hayes	Bootstrapped CIs	No
Lopez-Lopez (2014)—B	Pain (current, worst, least, and average pain combined into an overall 11-point NRS)	Pain interference (ARS)	Depression (GDS)	SEM	Bootstrapped CIs	No
Molton (2014)—A	Pain (11-point NRS)	Pain interference (PRO-PI-SF)	Depression (PHQ-9)	SEM	Bootstrapped CIs	No
Molton (2014)—B	Pain (11-point NRS)	Pain interference (PRO-PI-SF)	Depression (PHQ-9)	Baron and Kenny	Sobel	Yes
Molton (2014)—C	Pain (11-point NRS)	Pain interference (PRO-PI-SF)	Depression (PHQ-9)	Baron and Kenny	Sobel	Yes
Pjanic (2014)	Pain (100 mm VAS)	Social support (IRES 3.1); Self-efficacy (GSE)	Depression (HADS)	Baron and Kenny	NR	No
Rezaei (2014)	Depression (HADS Depression subscale)	Illness perception (IPQ)	Pain (RAPS)	Baron and Kenny	Sobel	Yes
Pakpour (2015)	Pain (100 mm VAS)	Sexual functioning (F: FSFI, M: IIEF)	Depression (HADS)	Baron and Kenny	Sobel	Yes
Peñacoba Puente (2015)	Pain (11-point NRS and PPTs)	Pain Self-Efficacy (CPSS); Positive and Negative Affects (PANAS)	Depression (HADS)	Baron and Kenny	Sobel and Bootstrapped CIs	Yes
Skidmore (2015)	Depression (BDI)	Pain Self-Efficacy (PSEQ)	Pain (MPQ-SF)	Baron and Kenny	Sobel	Yes
Wang (2015)—A	Pain (WOMAC pain subscale)	Physical function (WOMAC physical function subscale)	Depression (GDS)	SEM	Bootstrapped CIs	No
Wang (2015)—B	Pain (WOMAC pain subscale)	Physical function (WOMAC physical function subscale)	Depression (GDS)	Baron and Kenny	Sobel and Bootstrapped CIs	Yes
Wang (2015)—C	Pain (WOMAC pain subscale)	Physical function (WOMAC physical function subscale)	Depression (GDS)	Baron and Kenny	Sobel and Bootstrapped CIs	Yes
Wood (2016)	Pain (11-point NRS)	Helplessness (PRSS-C Helplessness subscale); Magnification (PRSS-C Magnification subscale)	Depression (DASS-21)	SEM	Bootstrapped CIs	Yes
Briest & Bethge (2017)	Depression (PHQ-9)	Pain catastrophizing (CSQ)	Pain (current, strongest, and average pain combined into an overall 11-point NRS)	Baron and Kenny	Sobel and Bootstrapped CIs	Yes
Müller (2017)—A	Pain (10-point NRS)	Participation restriction (USER-P Restriction subscale)	Depression (MHI-5 of SF-36)	Baron and Kenny	Sobel test	No
Müller (2017)—B	Pain (10-point NRS)	Participation satisfaction (USER-P—Satisfaction subscale)	Depression (MHI-5 of SF-36)	SEM	NR	No
Müller (2017)—C	Pain (10-point NRS)	Participation frequency (USER-P—Frequency subscale)	Depression (MHI-5 of SF-36)	SEM	NR	No
Cheng (2018)	Pain (CPG-P)	Pain catastrophizing (PCS)	Depression (CES-D)	SEM	NR	No
Lami (2018)	Pain (MPQ-SF)	Pain catastrophizing (PCS); Pain acceptance (CPAQ); Behavioral coping (COPE)	Depression (HADS)	Baron and Kenny	Bootstrapped CIs	Yes
Carvalho (2019)	Pain (11-point NRS)	Cognitive fusion (CFQ)	Depression (DASS-21)	Preacher and Hayes	Bootstrapped CIs	No
Costa (2019)	Pain (MPQ-SF)	Pain acceptance (CPAQ); Mindfulness (FFMQ)	Depression (DASS-42)	Preacher and Hayes	Bootstrapped CIs	No
Crawford (2019)—A	Depression (PHQ-9)	Pain catastrophizing (PCS); Emotion regulation (DERS)	Pain (MPQ-SF)	SEM	Bootstrapped CIs	No
Crawford (2019)—B	Pain (MPQ-SF)	Pain catastrophizing (PCS); Emotion regulation (DERS)	Depression (PHQ-9)	Preacher and Hayes	Bootstrapped CIs	Yes
Crawford (2019)—C	Depression (PHQ-9)	Helplessness (PCS-H); Rumination (PCS-R); Magnification (PCS-M); Emotion regulation (DERS)	Pain (MPQ-SF)	Preacher and Hayes	Bootstrapped CIs	Yes
Shigetoh (2019)	Depression (HADS)	Central sensitization (CSI)	Pain (MPQ-SF)	Preacher and Hayes	Bootstrapped CIs	Yes
Juan (2020)	Pain (100 mm VAS)	Sleep quality (PSQI); Exercise frequency (single question)	Depression (ZSDS)	Preacher and Hayes	Bootstrapped CIs	No
Sanchez-Rodrıguez (2020)—A	Pain (BPI)	Helplessness (PCS-H); pain-related cognitions (SOPA-35)	Depression (HSCL-20)	SEM	ML method	No
Sanchez-Rodrıguez (2020)—B	Depression (HSCL-20)	Helplessness (PCS-H); pain-related cognitions (SOPA-35)	Pain (BPI)	Preacher and Hayes	Bootstrapped CIs	Yes
Kim (2021)	Depression (PHQ-9)	Pain catastrophizing (PCS)	Pain (BPI-P)	SEM	NR	No
Li (2022)—A	Pain (11-point NRS)	Pain Interference (BPI-I)	Depression (PHQ-9)	SEM	Bootstrapped CIs	Yes
Li (2022)—B	Pain (11-point NRS)	Pain Interference (BPI-I)	Depression (PHQ-9)	SEM	Bootstrapped CIs	Yes
Li (2022)—C	Pain (11-point NRS)	Pain Interference (BPI-I)	Depression (PHQ-9)	SEM	Bootstrapped CIs	Yes
Nephew (2022)	Depression (PHQ-9)	Perceived stress (PSS); Sleep quality (PSQI); Pain self-efficacy (PSEQ)	Pain (BPI-P); Average pain in the last 7 days (BPI average pain item)	Baron and Kenny	NR	Yes
Paré (2023)	Depression (BDI-II)	Positive Outcome Expectancies (101-point NRS); Negative Outcome Expectancies (101-point NRS)	Pain (11-point NRS)	NR	NR	No
Kardash (2024)	Pain (BPI-P)	Pain catastrophizing (PCS)	Depression (DASS-21)	Preacher and Hayes	Bootstrapping CIs	Yes
Williams (2024)	Pain (ISCIPBD-P)	Quality of life (WHOQOL-BREF)	Depression (CES-D)	Preacher and Hayes	Bootstrapping CIs	No

Note: For studies reporting multiple mediation models, letters (A, B, C, etc.) are used solely to distinguish the different mediation models analyzed within the same study. Abbreviations: AIMS-2-PF, Arthritis Impact Management Scale 2–Physical Function subscale; AIMS-2-P, Arthritis Impact Management Scale 2–Pain subscale; ARS, Activity Restriction Scale; BDI, Beck Depression Inventory; BPI, Brief Pain Inventory; BPI-I, Brief Pain Inventory–Pain interference subscale; BPI-P, Brief Pain Inventory–Pain intensity subscale; CIs, Confidence Intervals; COPE Dispositional Questionnaire; CES-D, Center for Epidemiological Studies Depression Scale; CFQ, Cognitive Fusion Questionnaire; CPAQ, Chronic Pain Acceptance Questionnaire; CPG-P, Chronic Pain Grade–Pain intensity subscale; CPSS, Chronic Pain Self-Efficacy Scale; CSI, Central Sensitization Inventory; CSQ, Coping Strategies Questionnaire; CSQ-C, Coping Strategies Questionnaire–Catastrophizing subscale; DASS-21, Depression, Anxiety and Stress Scale–21 item version; DASS-42, Depression Anxiety and Stress Scale–42 item version; DERS, Difficulties in Emotion Regulation Scale; FABQ, Fear-Avoidance Beliefs Questionnaire; FIQ-P, Fibromyalgia Impact Questionnaire–Pain intensity item; FFMQ, Five Facet Mindfulness Questionnaire; FSFI, Female Sexual Function Index; GDS, Geriatric Depression Scale; GSE, General Self-Efficacy Scale; HADS, Hospital Anxiety and Depression Scale; HSCL-20, Hopkins Symptom Checklist; IIEF, International Index of Erectile Function; IPQ, Illness Perception Questionnaire; IRES, Indikatoren des Reha-Status; ISCIPBDS-P, International Spinal Cord Injury Pain Basic Data Set–Pain intensity item; KPI-B, Kiel Pain Inventory–Behavioral endurance subscale; KPI-C, Kiel Pain Inventory–Catastrophizing subscale; KPI-H, Kiel Pain Inventory–Help-/Hopelessness subscale; MAI-A, Multilevel Assessment Inventory–Activities subscales; MHI-5, 5-item Mental Health Index; ML, Maximum Likelihood; MPQ-P, McGill Pain Questionnaire–Pain rating Index; MPQ-SF, McGill Pain Questionnaire–Short Form; MSES, Moorong Self-Efficacy Scale; NR, Not Reported; NRS, Numerical Rating Scale; PANAS, Positive and Negative Affect Scale; PCS, Pain Catastrophizing Scale; PCS-H, Pain Catastrophizing Scale–Helplessness subscale; PCS-M, Pain Catastrophizing Scale–Magnification subscale; PCS-R, Pain Catastrophizing Scale–Rumination subscale; PGC-P, Philadelphia Geriatric Center–Pain scale; PHQ-9, Patient Health Questionnaire-9; POMS, Profile of Mood States, PPTs, Pressure Pain Thresholds; PRO-PI-FS, Measurement Information System (PROMIS) Pain Interference Short Form; PRSS-C, Pain-Related Self Statements Scale–Catastrophizing subscale; PSEQ, Pain Self-Efficacy Questionnaire; PSQI, Pittsburgh Sleep Quality Index; PSS, Perceived Stress Scale; RAI, Rheumatology Attitudes Index; RAPS, Rheumatoid Arthritis Pain Scale; SEM, Structural Equation Modeling; SF-36, 36-Item Short-Form Health Survey; SIGHD, Standardized Hamilton Rating Scale–Depression; SOPA-35, Survey of Pain Attitudes; TP, Tender Point; USER-P, Utrecht Scale of Evaluation of Rehabilitation–Participation; VAS, Visual Analog Scale; WOMAC, Western Ontario and McMaster University Osteoarthritis Index; WHOQOL-BREF, World Health Organization Quality of Life Brief Version; WHYMPI-I, West Haven–Yale Multidimensional Pain Inventory–Pain interference subscale; WHYMPI-P, West Haven–Yale Multidimensional Pain Inventory–Pain intensity subscale; ZSDS, Zung Self-rating Depression Scale.

**Table 3:** Methodological appraisal scores of the included mediation models.

First Author (Year)	Q1 ^1^	Q2 ^2^	Q3 ^3^	Q4 ^4^	Q5 ^5^	Q6 ^6^	Q7 ^7^	Q8 ^8^	Total Score
Bookwala (2003)	1	1	1	1	1	0	0	1	6
Klasen (2006)	1	1	0	1	1	0	0	0	4
Cannella (2007)	1	1	0	1	1	0	0	1	5
Palomino (2007)	1	1	0	1	1	0	0	1	5
Mirò (2011)	1	1	0	1	1	0	0	0	4
Fahland (2012)	1	1	1	1	1	0	0	0	5
Craig (2013)	0	1	0	1	0	0	0	0	2
Wood (2013)—A	1	1	1	1	1	0	0	0	5
Wood (2013)—B	1	1	0	1	1	0	0	0	4
Diaz-Piedra (2014)	1	1	0	1	1	0	0	0	4
Lopez-Lopez (2014)—A	1	1	0	1	1	0	0	0	4
Lopez-Lopez (2014)—B	1	1	0	1	1	0	0	0	4
Molton (2014)—A	1	1	0	1	1	0	0	1	5
Molton (2014)—B	1	1	1	1	1	0	0	1	6
Molton (2014)—C	1	1	0	1	1	0	0	1	5
Pjanic (2014)	0	1	1	1	NA *	0	1	1	5
Rezaei (2014)	1	1	0	1	1	0	0	1	5
Pakpour (2015)	1	1	1	1	1	0	0	1	6
Penacoba Puente (2015)	0	1	0	1	1	0	0	1	4
Skidmore (2015)	1	1	0	1	1	0	0	0	4
Wang (2015)—A	1	1	1	1	1	0	0	1	6
Wang (2015)—B	1	1	1	1	1	0	1	1	7
Wang (2015)—C	1	1	1	1	1	0	1	1	7
Wood (2016)	1	1	0	1	1	0	0	0	4
Briest (2017)	1	1	1	1	1	0	1	1	7
Muller (2017)—A	1	1	1	1	1	0	0	0	5
Muller (2017)—B	1	1	1	1	1	0	0	0	5
Muller (2017)—C	1	1	1	1	1	0	0	0	5
Cheng (2018)	1	1	1	1	1	0	0	1	6
Lami (2018)	1	1	0	1	1	0	0	0	4
Carvalho (2019)	1	1	1	1	1	0	0	0	5
Costa (2019)	1	1	0	1	1	0	0	0	4
Crawford (2019)—A	0	1	0	1	1	1	1	1	6
Crawford (2019)—B	0	1	0	1	1	1	1	1	6
Crawford (2019)—C	0	1	0	1	1	1	1	1	6
Shigetoh (2019)	0	1	0	1	1	0	0	0	3
Juan (2020)	1	1	1	1	1	0	0	0	5
Sanchez-Rodrıguez (2020)—A	1	1	1	1	1	0	0	1	6
Sanchez-Rodrıguez (2020)—B	1	1	1	1	1	0	0	1	6
Kim (2021)	1	1	0	1	1	0	0	1	5
Li (2022)—A	1	1	1	1	1	0	0	1	6
Li (2022)—B	1	1	1	1	1	0	0	1	6
Li (2022)—C	1	1	1	1	1	0	0	1	6
Nephew (2022)	0	1	1	1	1	1	1	0	5
Paré (2023)	1	1	1	1	1	0	1	1	6
Kardash (2024)	1	1	1	1	1	0	0	1	6
Williams (2024)	0	1	0	1	1	0	0	0	3

^1^ Did the study cite a theoretical or statistical framework supporting the proposed mediation hypothesis? (From a statistical perspective, the study must have reported empirical evidence of the relationship between X and Y, between X and M, and between M and Y.) ^2^ Is the study hypothesis/aim/objective clearly described? ^3^ Did the study use a sample that is adequate in terms of size or consistency with the research hypothesis? ^4^ Were the psychometric characteristics of the tools used to measure exposure, mediator, and outcome variables reported and were they within accepted ranges? (The study must have assessed the psychometric properties of the instrument within the investigated sample, reported its characteristics from other studies, provided references where such properties can be found, or used instruments that are widely recognized as valid and reliable (e.g., the Beck Depression Inventory).) ^5^ Were statistically appropriate/acceptable methods of data analysis used? ^6^ Did the study ascertain whether changes in the exposure variable preceded changes in the mediator variable? ^7^ Did the study ascertain whether changes in the mediator variable preceded changes in the outcome variable? ^8^ Did the study control for possible confounding factors? * Not applicable.

**Table 4:** Recommendations for future mediation research in chronic pain and depression.

Area	Recommendation
Study Design	Prefer longitudinal three-wave designs (T1–T2–T3) to establish temporal order of effects.
Confounding Control	Always control for key covariates (e.g., age, sex, SES, comorbidities, medications, baseline outcomes).
Sample Size	Ensure adequate statistical power through an *a priori* power analysis tailored to the specific mediation model, expected effect sizes, and study design.
Measurement	Use standardized and validated tools for predictors, mediators, and outcomes.
Reporting	Provide standardized and complete reporting (e.g., standardized β coefficients and standard errors for each path and effect).
Analytical Approach	Employ theory-driven, integrated models; consider multiple mediators and their interrelations.
Pain Duration	Explicitly assess and report pain duration to ensure relevance to CP population detection.

## Data Availability

The data presented in this study are available on reasonable request from the corresponding author.

## Supplementary Materials

The following supporting information can be downloaded at: https://www.mdpi.com/article/10.3390/jcm15155784/s1. Supplementary File S1. PRISMA 2020 Checklist; Supplementary File S2. PRISMA 2020 for Abstracts Checklist. Supplementary File S3: Search strategy for online consultation and search results. Supplementary File S4: R Script–Pain catastrophizing. Supplementary File S5: R Script–Helplessness. Supplementary File S6: Data used for the meta-analysis of pain catastrophizing: extracted regression coefficients (β) and standard errors. Supplementary File S7. Data used for the meta-analysis of helplessness: extracted regression coefficients (β) and standard errors. Supplementary File S8: Exploratory funnel plot for the meta-analysis of pain catastrophizing. Supplementary File S9: Exploratory funnel plot for the meta-analysis of helplessness.

## Abbreviations

The following abbreviations are used in this manuscript:

ACT	Acceptance and Commitment Therapy
BDI	Beck Depression Inventory
CBT	Cognitive-Behavioral Therapy
CBT-I	Cognitive-Behavioral Therapy–Insomnia
CES-D	Center for Epidemiological Studies Depression Scale
CINAHL	Cumulative Index to Nursing and Allied Health Literature
CP	Chronic Pain
CPG	Chronic Pain Grade
DASS	Depression, Anxiety and Stress Scale
DL	DerSimonian–Laird
GDS	Geriatric Depression Scale
HADS	Hospital Anxiety and Depression Scale
HRSD	Hamilton Rating Scale for Depression
HSCL	Hopkins Symptom Checklist
ISCIPBDS	International Spinal Cord Injury Pain Basic Data Set
MARS	Meta-Analysis Reporting Methods
NRS	Numerical Rating Scale
PHQ	Patient Health Questionnaire
POMS	Profile of Mood States
PRISMA	Preferred Reporting Items for Systematic Reviews and Meta-Analyses
PROSPERO	International Prospective Register of Systematic Reviews
RAPS	Rheumatoid Arthritis Pain Scale
RCTs	Randomized Controlled Trials
SE	Standard Error
SF-36	36-Item Short-Form Health Survey
SF-MPQ	Short Form of the McGill Pain Questionnaire
SWiM	Synthesis Without Meta-Analysis
TSSEM	Two-stage structural equation modeling
VAS	Visual Analog Scale
WHYMPI	West-Haven–Yale Multidimensional Pain Inventory
WOMAC	Western Ontario and McMaster University Osteoarthritis Index
ZSDS	Zung Self-rating Depression Scale
