# Supplementary material for: Mediators of the Pain–Depression Relationship in Adults with Chronic Pain: A Systematic Review and Exploratory Meta-Analyses
Source: J Clin Med. 2026 Jul 23;15(15):5784. doi: 10.3390/jcm15155784 (PMC13466972; doi:10.3390/jcm15155784)
Supplement: Supplementary file 1 [file jcm-15-05784-s001.zip › jcm-4402128-supplementary/Supplementary File S3 - Search Strategy for Online Consultation and Search Results.pdf]

Mediators of the Pain-Depression Relationship in Adults with Chronic Pain. A systematic review and meta-analysis.

Supplementary File S3. Search Strategy for Online Consultation and Search Results

PubMed/MEDline, Scopus, Web Of Science, CINAHL, Psychology Database, Cochrane Library, PsycINFO

Search Strategy for Online Consultation and Search Results

Review questions

This systematic review and meta-analysis aimed to:

1. identify factors mediating the effect of pain intensity on depressive symptoms, or vice versa, in adults with CP;
2. estimate the magnitude of indirect, direct and total effects reported across eligible studies.

Searches

- The core search filter will include the terms “pain”, “chronic pain”, “pain measurement”, “depression”, “depressive disorder”, mediat* OR indirect OR "structural equation modeling" OR "Baron and Kenny" OR MacKinnon OR "product of coefficient" OR "difference in coefficient" OR "process of change" OR sobel* OR "causal pathway".

Results

The present document includes the online search strategies from the online search run on **PubMed/MEDline, PubMed/MEDline, Scopus, Web Of Science, CINAHL, Psychology Database, Cochrane Library, PsycINFO.**

PubMed/MEDline

Online Search – 6 July 2023

SEARCH STRATEGY - PUBMED/MEDLINE

(((chronic pain OR pain OR pain measurement) AND (et [sh] OR px [sh] OR ep [sh] OR co [sh]))

AND ((depression OR depressive disorder) AND (px [sh] OR et [sh] OR co [sh])))

AND ((mediat* OR indirect OR "structural equation modeling" OR "structural equation modelling" OR "path" OR "Baron and Kenny" OR MacKinnon OR "product of coefficient" OR "difference in coefficient" OR "process of change" OR sobel* OR "causal pathway" OR intermediate OR "indirect effect" OR "process variable" OR "treatment effect" OR "process evaluation" OR mechanism OR SEM OR bootstrap* OR caus* OR "mediation stud*" OR mediation analysis))

Filters: Humans, Adult: 19+ years, from 2000 - 2023 Sort by: Most Recent

Results: 3116 citations retrieved

Scopus

Online Search – 12 June 2023

SEARCH STRATEGY – SCOPUS

(TITLE-ABS-KEY ((chronic AND pain) OR pain OR (pain AND measurement) OR "chronic pain") AND TITLE-ABS-KEY (((depression OR depressive) AND (disorder OR disease)) OR "depressive disorder" OR "depressive disease" OR depressed)

AND TITLE-ABS-KEY (mediat* OR indirect OR "structural equation modeling" OR "structural equation modelling" OR "path" OR "Baron and Kenny" OR mackinnon OR "product of coefficient" OR "difference in coefficient" OR "process of change" OR sobel* OR "causal pathway" OR intermediate OR "indirect effect" OR "process variable" OR "treatment effect" OR "process evaluation" OR mechanism OR sem OR bootstrap* OR caus* OR "mediation stud*" OR (mediation AND analysis)))

AND (TITLE (pain AND depress*))

AND (LIMIT-TO (PUBYEAR , 2023) OR LIMIT-TO (PUBYEAR , 2022) OR LIMIT-TO (PUBYEAR , 2021) OR LIMIT-TO (PUBYEAR , 2020) OR LIMIT-TO (PUBYEAR , 2019) OR LIMIT-TO (PUBYEAR , 2018) OR LIMIT-TO (PUBYEAR , 2017) OR LIMIT-TO (PUBYEAR , 2016) OR LIMIT-TO (PUBYEAR , 2015) OR LIMIT-TO (PUBYEAR , 2014) OR LIMIT-TO (PUBYEAR , 2013) OR LIMIT-TO (PUBYEAR , 2012) OR LIMIT-TO (PUBYEAR , 2011) OR LIMIT-TO (PUBYEAR , 2010) OR LIMIT-TO (PUBYEAR , 2009) OR LIMIT-TO (PUBYEAR , 2008) OR LIMIT-TO (PUBYEAR , 2007) OR LIMIT-TO (PUBYEAR , 2006) OR LIMIT-TO (PUBYEAR , 2005) OR LIMIT-TO (PUBYEAR , 2004) OR LIMIT-TO (PUBYEAR , 2003) OR LIMIT-TO (PUBYEAR , 2002) OR LIMIT-TO (PUBYEAR , 2001) OR LIMIT-TO (PUBYEAR , 2000))

AND (LIMIT-TO (EXACTKEYWORD , "Human"))

Results: 460 citations retrieved

Web of Science

Online Search – 13 June 2023

SEARCH STRATEGY – WEB OF SCIENCE

(chronic pain OR pain OR pain measurement) AND (etiol* OR etiologic OR epidemiol* OR epidemiol* OR complicat*) (Topic)

AND (depression OR "depressive disorder" OR "depressive disease" OR depressed) AND (etiol* OR etiologic OR epidemiol* OR epidemiol* OR complicat*) (Topic)

AND mediat* OR indirect OR "structural equation modeling" OR "structural equation modelling" OR "path" OR "Baron and Kenny" OR MacKinnon OR "product of coefficient" OR "difference in coefficient" OR "process of change" OR sobel* OR "causal pathway" OR intermediate OR "indirect effect" OR "process variable" OR "treatment effect" OR "process evaluation" OR mechanism OR SEM OR bootstrap* OR caus* OR "mediation stud*" OR mediation analysis (Topic)

AND depress* OR pain (Title)

AND 2023 or 2022 or 2021 or 2020 or 2019 or 2018 or 2017 or 2016 or 2015 or 2014 or 2013 or 2012 or 2011 or 2010 or 2009 or 2008 or 2007 or 2006 or 2005 or 2004 or 2003 or 2002 or 2001 or 2000 (Publication Years)

Results: 613 citations retrieved

CINAHL

Online Search – 13 June 2023

SEARCH STRATEGY – CINAHL

TI(chronic N1 pain) OR AB(chronic N1 pain) OR TI (pain N1 measurement) OR MM(“chronic pain” OR “pain”) OR MH(“chronic pain”)

AND MM(“depression” OR TI(depressive N1 disorder) OR AB(depressive N1 disorder) OR MH (“depression” OR “depressive disorder”)

AND (mediat* OR indirect OR "structural equation modeling" OR "structural equation modelling" OR "path" OR "Baron and Kenny" OR mackinnon OR "product of coefficient" OR "difference in coefficient" OR "process of change" OR sobel* OR "causal pathway" OR intermediate OR "indirect effect" OR "process variable" OR "treatment effect" OR "process evaluation" OR mechanism OR sem OR bootstrap* OR caus* OR "mediation stud*" OR TI(mediation N1 analysis) OR AB(mediation N1 analysis)

Results: 83 citations retrieved

Psychology Database (ProQuest)

Online Search – 9 May 2023

SEARCH STRATEGY – PSYCHOLOGY DABATASE

((((((chronic pain OR pain OR pain measurement) AND (et [sh] OR px [sh] OR ep [sh] OR co [sh]))

AND ((depression OR depressive disorder) AND (px [sh] OR et [sh] OR co [sh])))

AND ((mediat* OR indirect OR "structural equation modeling" OR "structural equation modelling" OR "path" OR "Baron and Kenny" OR MacKinnon OR "product of coefficient" OR "difference in coefficient" OR "process of change" OR sobel* OR "causal pathway" OR intermediate OR "indirect effect" OR "process variable" OR "treatment effect" OR "process evaluation" OR mechanism OR SEM OR bootstrap* OR caus* OR "mediation stud*" OR mediation analysis)))

AND humans)

AND YR(>2000))

NOT ((rtype(letter) OR mesh("letter") OR rtype(editorial) OR rtype(historical article) OR rtype(commentary) OR rtype(note) OR mesh("case report")) OR (rtype(case PRE/0 report?) OR mesh("case study") OR rtype("case study") OR mesh("animals, laboratory") OR mesh("models, animal")) OR (su("Non-human") OR su(animal experimentation) OR animal PRE/3 experiment OR su(animal PRE/0 model) OR mesh("rodentia")) OR (su(rodent OR rodents) OR su(laboratory PRE/3 animals) OR mesh("Observational Studies, Veterinary as Topic" OR mice OR mouse OR conference OR proceedings OR dissertation))))

AND (ti,ab(pain*) AND ti,ab(depress*))

Results: 294 citations retrieved

Cochrane Library

Online Search – 13 June 2023

SEARCH STRATEGY – COCHRANE LIBRARY

(“chronic pain”

AND depression OR depressed) (in Title Abstract Keyword)

NOT covid (in Title Abstract Keyword)

Results: 2201 Trials retrieved

PsycINFO

Online Search – 6 July 2023

SEARCH STRATEGY – PsycINFO

TI(chronic N1 pain) OR AB(chronic N1 pain) OR TI (pain N1 measurement) OR MM(“chronic pain” OR “pain”) OR MH(“chronic pain”)

AND MM(“depression” OR TI(depressive N1 disorder) OR AB(depressive N1 disorder) OR MH (“depression” OR “depressive disorder”)

AND (mediat* OR indirect OR "structural equation modeling" OR "structural equation modelling" OR "path" OR "Baron and Kenny" OR mackinnon OR "product of coefficient" OR "difference in coefficient" OR "process of change" OR sobel* OR "causal pathway" OR intermediate OR "indirect effect" OR "process variable" OR "treatment effect" OR "process evaluation" OR mechanism OR sem OR bootstrap* OR caus* OR "mediation stud*" OR TI(mediation N1 analysis) OR AB(mediation N1 analysis)

Filters: Humans; Adult: 18+ years; from 2000 – 2023; Language: English, Italian, German; Exclude dissertations

Results: 59 citations retrieved

PubMed/MEDline

Online Search Update – 17 May 2024

SEARCH STRATEGY - PUBMED/MEDLINE – 17 MAY 2024

(((chronic pain OR pain OR pain measurement) AND (et [sh] OR px [sh] OR ep [sh] OR co [sh]))

AND ((depression OR depressive disorder) AND (px [sh] OR et [sh] OR co [sh])))

AND ((mediat* OR indirect OR "structural equation modeling" OR "structural equation modelling" OR "path" OR "Baron and Kenny" OR MacKinnon OR "product of coefficient" OR "difference in coefficient" OR "process of change" OR sobel* OR "causal pathway" OR intermediate OR "indirect effect" OR "process variable" OR "treatment effect" OR "process evaluation" OR mechanism OR SEM OR bootstrap* OR caus* OR "mediation stud*" OR mediation analysis))

Filters: Humans, Adult: 19+ years, from 31 January 2023 onwards Sort by: Most Recent

Results: 154 citations retrieved

SCOPUS

Online Search Update – 20th May 2024

SEARCH STRATEGY – CHRONIC PAIN AND DEPRESSION – SCOPUS 20 MAY 2023

(TITLE-ABS-KEY (mediat* OR indirect OR "structural equation modeling" OR "structural equation modelling" OR "path" OR "Baron and Kenny" OR mackinnon OR "product of coefficient" OR "difference in coefficient" OR "process of change" OR sobel* OR "causal pathway" OR intermediate OR "indirect effect" OR "process variable" OR "treatment effect" OR "process evaluation" OR mechanism OR sem OR bootstrap* OR caus* OR "mediation stud*" OR (mediation AND analysis))) AND (TITLE-ABS-KEY ((chronic AND pain) OR pain OR (pain AND measurement) OR "chronic pain")) AND (TITLE-ABS-KEY (((depression OR depressive) AND (disorder OR disease)) OR "depressive disorder" OR "depressive disease" OR depressed)) AND ((TITLE (pain AND depress*))) AND (LIMIT-TO (PUBYEAR , 2023) OR LIMIT-TO (PUBYEAR , 2024)) AND (LIMIT-TO (LANGUAGE , "English") OR LIMIT-TO (LANGUAGE , "Italian") OR LIMIT-TO (LANGUAGE , "German") OR LIMIT-TO (LANGUAGE , "French") OR LIMIT-TO (LANGUAGE , "Spanish"))

Results: 81 citations retrieved

WEB OF SCIENCE

Online Search Update – 23rd May 2024

Search Strategy – Chronic pain and depression – Web of Science 23 May 2023

#1 (chronic pain OR pain OR pain measurement) AND (etiol* OR etiologic OR epidemiol* OR epidemiol* OR complicat*) (Topic)

AND

#2 TS=((depression OR "depressive disorder" OR "depressive disease" OR depressed) AND (etiol* OR etiologic OR epidemiol* OR epidemiol* OR complicat*)

AND

#3 (TS=(mediat* OR indirect OR "structural equation modeling" OR "structural equation modelling" OR "path" OR "Baron and Kenny" OR MacKinnon OR "product of coefficient" OR "difference in coefficient" OR "process of change" OR sobel* OR "causal pathway" OR intermediate OR "indirect effect" OR "process variable" OR "treatment effect" OR "process evaluation" OR mechanism OR SEM OR bootstrap* OR caus* OR "mediation stud*" OR mediation analysis (Topic)))

AND

#4 TI=(depress* OR pain)

#5 #4 AND #3 AND #2 AND #1

#6 #4 AND #3 AND #2 AND #1 and 2024 or 2023 (Publication Years)

#7 #4 AND #3 AND #2 AND #1 and 2024 or 2023 (Publication Years) and English (Languages)

Results: 74 citations retrieved

CINHAL

Online Search Update – 10th Jun 2024

Search Strategy – Chronic pain and depression – CINHAL 10 June 2024

#1 TI(chronic N1 pain) OR AB(chronic N1 pain) OR TI (pain N1 measurement) OR MM(“chronic pain” OR “pain”) OR MH(“chronic pain”)

AND

#2 MM(“depression” OR TI(depressive N1 disorder) OR AB(depressive N1 disorder) OR MH (“depression” OR “depressive disorder”)

AND

#3 (mediat* OR indirect OR "structural equation modeling" OR "structural equation modelling" OR "path" OR "Baron and Kenny" OR mackinnon OR "product of coefficient" OR "difference in coefficient" OR "process of change" OR sobel* OR "causal pathway" OR intermediate OR "indirect effect" OR "process variable" OR "treatment effect" OR "process evaluation" OR mechanism OR sem OR bootstrap* OR caus* OR "mediation stud*" OR TI(mediation N1 analysis) OR AB(mediation N1 analysis)

Limiters: Publication Date: 20230101-20241231; Exclude MEDLINE records; Human

Expanders: Apply equivalent subjects

Search modes: Boolean/Phrase

Results: 85 citations retrieved

Psychology Database (ProQuest)

Online Search Update – 10 Jun 2024

SEARCH STRATEGY – PSYCHOLOGY DABATASE – 10 JUNE 2024

(((((((chronic pain OR pain OR pain measurement) AND (et [sh] OR px [sh] OR ep [sh] OR co [sh]))

AND

((depression OR depressive disorder) AND (px [sh] OR et [sh] OR co [sh])))

AND

((mediat* OR indirect OR "structural equation modeling" OR "structural equation modelling" OR "path" OR "Baron and Kenny" OR MacKinnon OR "product of coefficient" OR "difference in coefficient" OR "process of change" OR sobel* OR "causal pathway" OR intermediate OR "indirect effect" OR "process variable" OR "treatment effect" OR "process evaluation" OR mechanism OR SEM OR bootstrap* OR caus* OR "mediation stud*" OR mediation analysis)))

AND humans)

AND LA(english))

NOT ((rtype(letter) OR mesh("letter") OR rtype(editorial) OR rtype(historical article) OR rtype(commentary) OR rtype(note) OR mesh("case report")) OR (rtype(case PRE/0 report?) OR mesh("case study") OR rtype("case study") OR mesh("animals, laboratory") OR mesh("models, animal")) OR (su("Non-human") OR su(animal experimentation) OR animal PRE/3 experiment OR su(animal PRE/0 model) OR mesh("rodentia")) OR (su(rodent OR rodents) OR su(laboratory PRE/3 animals) OR mesh("Observational Studies, Veterinary as Topic" OR mice OR mouse OR conference OR proceedings OR dissertation))))

AND (ti,ab(pain*) AND ti,ab(depress*)))

AND pd(20230501-20241231)

Results: 20 citations retrieved

Cochrane Library

Online Search Update – 10 Jun 2024

SEARCH STRATEGY – COCHRANE LIBRARY

(“chronic pain”

AND depression OR depressed) (in Title Abstract Keyword)

NOT covid (in Title Abstract Keyword)

Results: 7 Trials retrieved

PsycINFO

Online Search Update – 17 May 2024

SEARCH STRATEGY – PsycINFO

TI(chronic N1 pain) OR AB(chronic N1 pain) OR TI (pain N1 measurement) OR MM(“chronic pain” OR “pain”) OR MH(“chronic pain”)

AND MM(“depression” OR TI(depressive N1 disorder) OR AB(depressive N1 disorder) OR MH (“depression” OR “depressive disorder”)

AND (mediat* OR indirect OR "structural equation modeling" OR "structural equation modelling" OR "path" OR "Baron and Kenny" OR mackinnon OR "product of coefficient" OR "difference in coefficient" OR "process of change" OR sobel* OR "causal pathway" OR intermediate OR "indirect effect" OR "process variable" OR "treatment effect" OR "process evaluation" OR mechanism OR sem OR bootstrap* OR caus* OR "mediation stud*" OR TI(mediation N1 analysis) OR AB(mediation N1 analysis)

Filters: Humans; Adult: 18+ years; from 1 July 2023 onwards; Language: English, Italian, German; Exclude dissertations

Results: 2 citations retrieved
