# Supplementary material for: Mediators of the Pain–Depression Relationship in Adults with Chronic Pain: A Systematic Review and Exploratory Meta-Analyses
Source: J Clin Med. 2026 Jul 23;15(15):5784. doi: 10.3390/jcm15155784 (PMC13466972; doi:10.3390/jcm15155784)
Supplement: Supplementary file 1 [file jcm-15-05784-s001.zip › jcm-4402128-supplementary/Supplementary File S4 - R Script - Pain catastrophizing.pdf]

```
########################################################
# Supplementary File S4
#
# R script for the meta-analysis of pain catastrophizing
#
# Input file:
# Supplementary_File_S6_Catastrophizing_Data.csv
########################################################

library(metafor)

mydata<-read.csv("Supplementary_File_S6_Catastrophizing_Data.csv",
header=TRUE, sep=";", dec=",")

lab<-as.vector(mydata$Studies)

###############################
# INDIRECT EFFECT
###############################

res2 <- rma(yi = betastand_indirect, sei = SE_indirect,
slab=paste(lab, Year, sep=", "),data = mydata, method = "DL")

summary(res2)

forest(res2, level=95, refline=0,xlab = "Effect Size",weight =
TRUE,showweights=TRUE, header=TRUE, top=3, main ="Catastrophizing
for Indirect Effect")

funnel(res2)

loo_results2 <- leave1out(res2)

# Print results

print(loo_results2)

forest(loo_results2$estimate, ci.lb=loo_results2$ci.lb,
order="prec",showweights=TRUE, header=TRUE, top=3, main
="Catastrophizing for Indirect Effect", ci.ub=loo_results2$ci.ub,
xlab="Effect Size",  slab=paste("Without Study",lab,
rownames(loo_results2)))

###############################
# TOTAL EFFECT
###############################
res3 <- rma(yi = betastand_total, sei = SE_Total, slab=paste(lab,
Year, sep=", "),data = mydata, method = "DL")
```

```
summary(res3)


forest(res3, level=95, refline=0,xlab = "Effect Size",
header=TRUE, top=3, weight = TRUE, main ="Catastrophizing for Total
Effect")


funnel (res3)


loo_results3 <- leave1out(res3)

# Print results

print(loo_results3)


forest(loo_results3$estimate, ci.lb=loo_results3$ci.lb,
order="prec",showweights=TRUE, header=TRUE, top=3, main
="Catastrophizing for Total Effect", ci.ub=loo_results3$ci.ub,
xlab="Effect Size",  slab=paste("Without Study",lab,
rownames(loo_results3)))
```

```
###############################
# DIRECT EFFECT
###############################
res4 <- rma(yi = betastand_direct, sei = SE_direct,
slab=paste(lab, Year, sep=", "),data = mydata, method = "DL")

summary(res4)


forest(res4, level=95, refline=0,xlab = "Effect Size",
showweights=TRUE, header=TRUE, top=3, main ="Catastrophizing for
Direct Effect")

funnel(res4)

loo_results4 <- leave1out(res4)

# Print results

print(loo_results4)


forest(loo_results4$estimate, ci.lb=loo_results4$ci.lb,
showweights=TRUE, header=TRUE, top=3, main ="Catastrophizing for
```

```
Direct Effect", ci.ub=loo_results4$ci.ub, xlab="Effect Size",
slab=paste("Without Study",lab, rownames(loo_results4)))
```
