# Supplementary material for: Mediators of the Pain–Depression Relationship in Adults with Chronic Pain: A Systematic Review and Exploratory Meta-Analyses
Source: J Clin Med. 2026 Jul 23;15(15):5784. doi: 10.3390/jcm15155784 (PMC13466972; doi:10.3390/jcm15155784)
Supplement: Supplementary file 1 [file jcm-15-05784-s001.zip › jcm-4402128-supplementary/Supplementary File S5 - R Script - Helplessness.pdf]

```
########################################################
# Supplementary File S5
#
# R script for the meta-analysis of Helplessness
#
# Input file:
# Supplementary_File_S7_Helplessness_Data.csv
########################################################


library(metafor)


mydata<-read.csv("Supplementary_File_S7_Helplessness_Data.csv",
header=TRUE, sep=";", dec=",")

lab<-as.vector(mydata$Studies)


###############################
# DIRECT EFFECT
###############################
res5 <- rma (yi = betastand_direct, sei = SE_direct,
slab=paste(lab, Year, sep=", "),data = mydata, method="DL")


summary(res5)


forest(res5, level=95, refline=0,xlab = "Effect Size", weight =
TRUE, showweights=TRUE, header=TRUE, top=3, main ="Helplessness
for Direct Effect")


funnel (res5)


loo_results5 <- leave1out(res5)

# Print results

print(loo_results5)


forest(loo_results5$estimate, ci.lb=loo_results5$ci.lb,
order="prec",showweights=TRUE, header=TRUE, top=3, main ="
Helplessness for Direct Effect", ci.ub=loo_results5$ci.ub,
xlab="Effect Size",  slab=paste("Without Study",lab,
rownames(loo_results5)))
```

```
################################
# TOTAL EFFECT
################################


res6 <- rma (yi = betastand_total, sei = SE_Total, slab=paste(lab,
Year, sep=", "),data = mydata, method="DL")


summary(res6)




forest(res6, level=95, refline=0,xlab = "Effect Size",
showweights=TRUE, header=TRUE, top=3, main ="Helplessness for
Total Effect")




funnel (res6)




loo_results6 <- leave1out(res6)

# Print results

print(loo_results6)


forest(loo_results6$estimate, ci.lb=loo_results6$ci.lb,
order="prec",showweights=TRUE, header=TRUE, top=3, main ="
Helplessness for Total Effect", ci.ub=loo_results6$ci.ub,
xlab="Effect Size",  slab=paste("Without Study",lab,
rownames(loo_results6)))




################################
# INDIRECT EFFECT
################################
res7 <- rma(yi = betastand_indirect, sei = SE_indirect,
slab=paste(lab, Year, sep=", "),data = mydata, method = "DL")

summary(res7)
```

```
forest(res7, level=95, refline=0,xlab = "Effect Size",
showweights=TRUE, header=TRUE, top=3, main ="Helplessness for
Indirect Effect")


funnel(res7)


loo_results7 <- leave1out(res7)

# Print results

print(loo_results7)



forest(loo_results7$estimate, ci.lb=loo_results7$ci.lb,
order="prec",showweights=TRUE, header=TRUE, top=3, main ="
Helplessness for Indirect Effect", ci.ub=loo_results7$ci.ub,
xlab="Effect Size",  slab=paste("Without Study",lab,
rownames(loo_results7)))
```
