# Supplementary material for: Mediators of the Pain–Depression Relationship in Adults with Chronic Pain: A Systematic Review and Exploratory Meta-Analyses
Source: J Clin Med. 2026 Jul 23;15(15):5784. doi: 10.3390/jcm15155784 (PMC13466972; doi:10.3390/jcm15155784)
Supplement: Supplementary file 1 [file jcm-15-05784-s001.zip › jcm-4402128-supplementary/Supplementary File S9 - Exploratory funnel plot for the meta-analysis of helplessness.pdf]

Supplementary File S8. Exploratory funnel plots for the meta-analysis of helplessness

Funnel plots of the total, direct, and indirect effects included in the meta-analysis of helplessness. Because only four studies were available for each quantitative synthesis, publication bias was not formally assessed. These funnel plots are provided for transparency and exploratory purposes only and should be interpreted with caution.

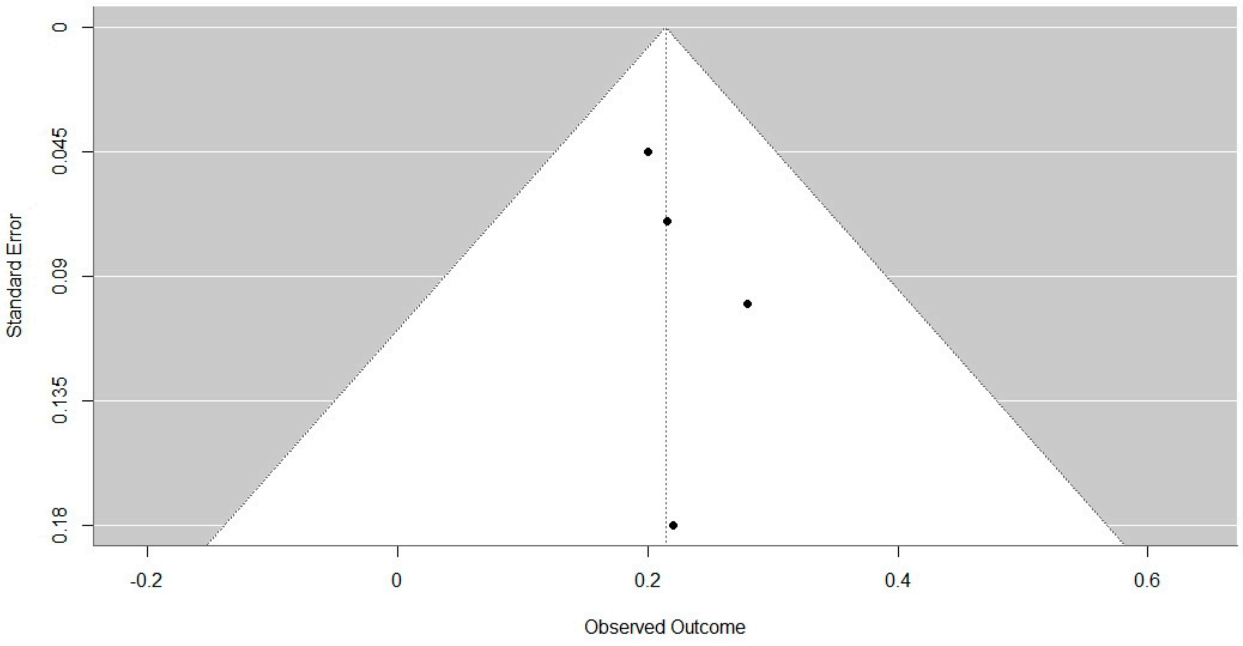

Figure S1. Funnel plot for the total effect.

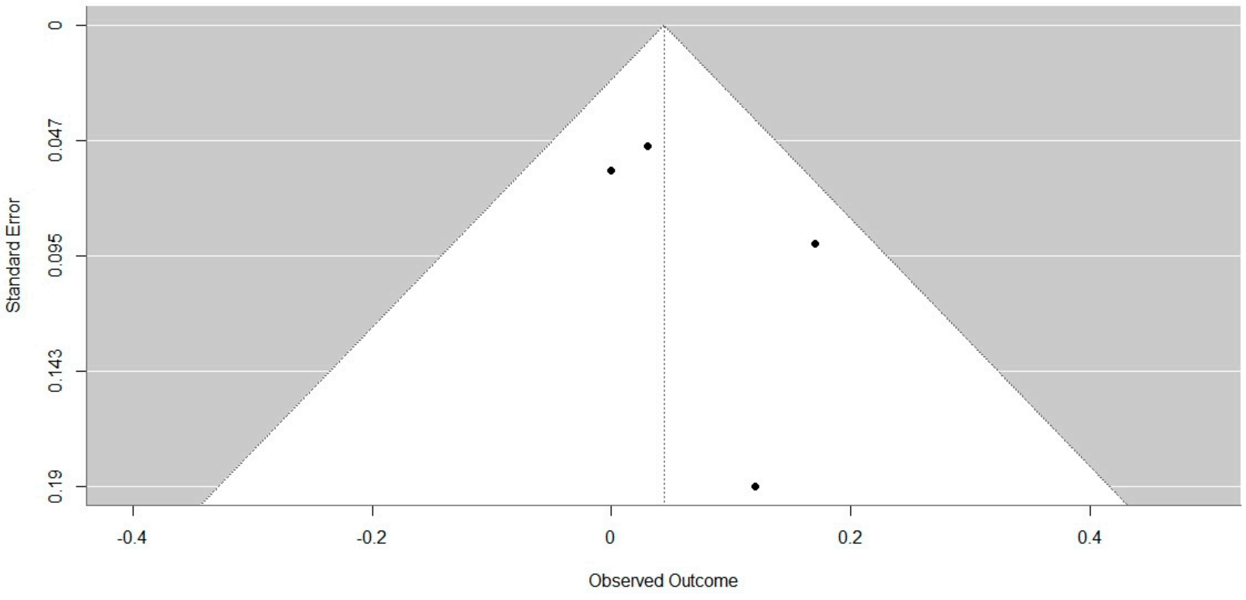

Figure S2. Funnel plot for the direct effect.

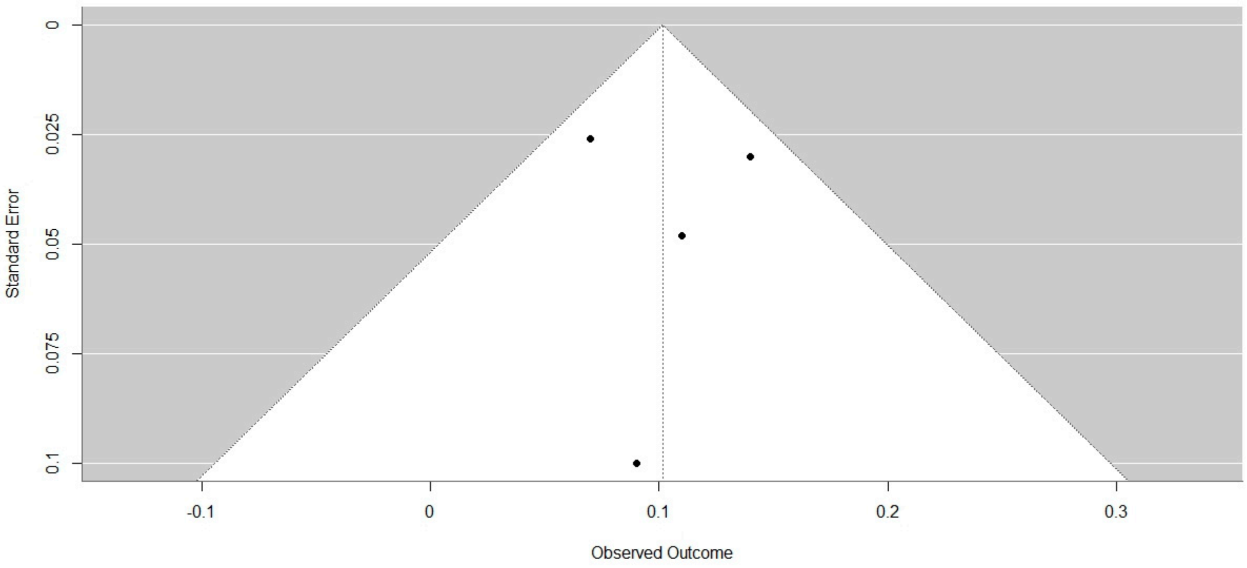

Figure S3. Funnel plot for the indirect effect.
